# Identification of key genes and molecular pathways regulating heat stress tolerance in pearl millet to sustain productivity in challenging ecologies

**DOI:** 10.3389/fpls.2024.1443681

**Published:** 2024-08-22

**Authors:** Swati Singh, Aswini Viswanath, Animikha Chakraborty, Neha Narayanan, Renuka Malipatil, Jinu Jacob, Shikha Mittal, Tara C. Satyavathi, Nepolean Thirunavukkarasu

**Affiliations:** ^1^ Genomics and Molecular Breeding Lab, Global Center of Excellence on Millets (Shree Anna), ICAR-Indian Institute of Millets Research, Hyderabad, India; ^2^ Department of Biotechnology and Bioinformatics, Jaypee University of Information Technology, Waknaghat, Solan, India

**Keywords:** abiotic stress, climate resilience, functional genes, heat stress, RNAseq, pearl millet, transcriptomes

## Abstract

Pearl millet is a nutri-cereal that is mostly grown in harsh environments, making it an ideal crop to study heat tolerance mechanisms at the molecular level. Despite having a better-inbuilt tolerance to high temperatures than other crops, heat stress negatively affects the crop, posing a threat to productivity gain. Hence, to understand the heat-responsive genes, the leaf and root samples of two contrasting pearl millet inbreds, EGTB 1034 (heat tolerant) and EGTB 1091 (heat sensitive), were subjected to heat-treated conditions and generated genome-wide transcriptomes. We discovered 13,464 differentially expressed genes (DEGs), of which 6932 were down-regulated and 6532 up-regulated in leaf and root tissues. The pairwise analysis of the tissue-based transcriptome data of the two genotypes demonstrated distinctive genotype and tissue-specific expression of genes. The root exhibited a higher number of DEGs compared to the leaf, emphasizing different adaptive strategies of pearl millet. A large number of genes encoding ROS scavenging enzymes, WRKY, NAC, enzymes involved in nutrient uptake, protein kinases, photosynthetic enzymes, and heat shock proteins (HSPs) and several transcription factors (TFs) involved in cross-talking of temperature stress responsive mechanisms were activated in the stress conditions. Ribosomal proteins emerged as pivotal hub genes, highly interactive with key genes expressed and involved in heat stress response. The synthesis of secondary metabolites and metabolic pathways of pearl millet were significantly enriched under heat stress. Comparative synteny analysis of HSPs and TFs in the foxtail millet genome demonstrated greater collinearity with pearl millet compared to proso millet, rice, sorghum, and maize. In this study, 1906 unannotated DEGs were identified, providing insight into novel participants in the molecular response to heat stress. The identified genes hold promise for expediting varietal development for heat tolerance in pearl millet and similar crops, fostering resilience and enhancing grain yield in heat-prone environments.

## Introduction

Pearl millet [*Pennisetum glaucum (L.R. Br)*] belongs to the Poaceae family. It is a crop widely grown in the arid and semi-arid regions of Sub-Saharan Africa and the Indian subcontinent, where other cereals fail to achieve an economic yield (Sun et al., 2020). India is the world’s largest pearl millet producer, with a total cultivated area of 7.41 million hectares and a production output of 10.3 million tons in 2020-2021 (Indiastat, 2020). It is a hardy crop that can withstand the unpredictable effects of climate change. Climate change endangers agricultural production, raising serious worries about global food security. Temperature fluctuations are a significant component that significantly impacts crop growth and development. Climate models predict that the production of pearl millet in Sub-Saharan Africa will reduce from 17% to 7% by 2050 (Schlenker and David, 2010).

Heat stress is a major environmental threat that reduces crop productivity and results in yield reduction (Jagadish et al., 2021). In comparison to other crops, pearl millet has a high tolerance level to abiotic stresses such as heat, drought, salinity, and nutrient deficiency (Vadez et al., 2012), which allows it to produce higher yields under the same conditions and is critical in ensuring food and nutritional security in fluctuating climatic conditions. It is a climate-resilient crop that can survive high temperatures of up to 42°C; however, when exposed to temperatures exceeding 42°C, crop carbohydrate reserves are depleted, and plant starvation occurs (Djanaguiraman et al., 2009). Furthermore, it results in decreased growth due to a loss of cellular water content and an overall reduction in cell size. This crop struggles to survive under prolonged heat stress and suffers from adverse effects such as compromised cell membrane integrity, a significant drop in chlorophyll content, and a decrease in antioxidant enzymes, resulting in the accumulation of free radicals that cause cell damage and apoptosis (Hasanuzzaman et al., 2013). Therefore, it is imperative to develop heat-tolerant varieties that can endure changes caused by high temperatures in the production ecologies in which they are cultivated.

Studies have revealed that pearl millet has outperformed maize regarding morphological and physiological indices such as relative growth rate and net assimilation rate (NAR) at high temperatures (Ashraf and Hafeez, 2004). The molecular mechanism entails the activation of transcription factors (TFs), heat shock proteins (HSPs), metabolite synthesis, and other heat stress-related genes (He et al., 2023). The repository of genes in pearl millet distinguishes it from other crops. Mwadzingeni et al., 2016 described heat stress as a complicated process mediated by an intricate interplay of many genes and their regulated expression (Mwadzingeni et al., 2016). Transcriptome analysis has emerged as a valuable methodology to investigate gene expression and complex regulatory networks. Its application has been beneficial in unravelling the molecular mechanisms operative in crops when exposed to heat stress (Frey et al., 2015). Recent studies have effectively used transcriptome-based approaches in several crops to elucidate the molecular function of abiotic stress tolerance namely, maize (Qian et al., 2019), rice (Wang et al., 2019), wheat (Rangan et al., 2020), eggplant (Zhang et al., 2019) and pearl millet (Goud et al., 2022).

Pearl millet genotypes show a wide level of variation in heat tolerance when compared to other cereal crops. The present study was designed to mine the heat-stress-responsive genes from such genetic variation. Here, two pearl millet inbreds contrasting to heat tolerance behavior were used to discover DEGs through a genome-wide RNA-Seq approach. We discovered DEGs that encode important transcription factors, ion transporters, and metabolic pathway regulators from the leaf and root tissues. Our findings establish the groundwork for mining essential genes linked with heat tolerance in pearl millet and elucidating the molecular mechanisms operating in pearl millet in response to heat stress. It will also provide valuable insights into improving pearl millet productivity under heat-stress ecologies in the changing climate scenarios.

## Materials and methods

### Plant materials and treatment condition

The study used two contrasting pearl millet genotypes, namely EGTB 1034 (heat-tolerant) and EGTB 1091 (heat-sensitive), developed from the pearl millet breeding program at ICAR-IIMR, Hyderabad. Several genotypes were systematically evaluated under heat stress in both pre-and post-flowering stages, and the above-contrasting genotypes were selected based on phenotypic performances for further characterization through transcriptomes. The seeds of these genotypes were grown in cups under a photoperiod of 16 hours of light and 8 hours of darkness at room temperature with 90% relative humidity. Heat stress was induced on seven-day-old seedlings in a controlled growth chamber with a constant temperature of 45°C for 24 hours, whereas, for control samples, the same procedure was performed at room temperature 30°C. Immediately after 24 hours, leaf and root samples were collected with three biological replicates from both control and heat-treated conditions of HTG and HSG and used for RNA sequencing, independently.

### RNA extraction and library preparation

Total RNA from the leaf and root of the three biological replicates of both control and treated genotypes was extracted using TRIzol reagent (RNAiso-plus) (ThermoFisher Scientific, United States), following the protocol of the manufacturer and further purified using MN Nucleospin RNA clean up kit (Macherey-Nagel, Germany). RNA quality and integrity were checked in 1% agarose (Lonza, Belgium) and Nanodrop 2000 (Thermofisher Scientific, Massachusetts, USA). Furthermore, the samples with RNA integrity number ≥7 were processed for analysis (Hosseini et al., 2021). The libraries were constructed using the KAPA HyperPrep Kit for the cDNA Synthesis & Amplification module, and length assessment was carried out using a bioanalyzer (Agilent Technologies, Santa Clara, California, USA). Then, the RNA-Seq libraries were sequenced on the Illumina sequencing platform (NovaSeq 6000) using a paired-end approach.

### Transcriptome sequencing analysis

Following RNA-Seq, a quality assessment of the reads was performed using FastQC (http://www.bioinformatics.babraham.ac.uk/projects/fastqc/), and adapter sequences and Illumina-specific sequences were removed using Trimmomatic (Version 0.36) (Bolger et al., 2014). The remaining cleaned reads were mapped to the reference genome of pearl millet (**Table 1**), obtained from the International Pearl Millet Genome Sequence Consortium (https://cegsb.icrisat.org/ipmgsc/genome.html) using the alignment tool Hisat2 ver. 2.0.4. Finally, the number of reads for each gene was counted using the featureCounts tool (version 2.0.0) (Liao et al., 2014).

**Table 1:** Summary of RNA-Seq data sets acquired from 24 samples of heat-tolerant and sensitive genotypes under heat stress conditions mapped to pearl millet reference genome.

Sample ID	Tissue	Conditions	Raw reads	Aligned reads	Alignment rate	Uniquely mapped reads (%)	Multi-mapped reads (%)
HTG_CL_R1	Leaf	Control	5.00E+07	42329687	82.46%	53.22%	29.24%
HTG_CR_R1	Root	Control	5.00E+07	32191133	68.98%	50.08%	18.90%
HTG_CL_R2	Leaf	Control	3.00E+07	20003729	71.83%	48.25%	23.58%
HTG_CR_R2	Root	Control	3.00E+07	24828065	71.27%	51.00%	20.27%
HTG_CL_R3	Leaf	Control	5.00E+07	38766968	79.55%	46.34%	33.21%
HTG_CR_R3	Root	Control	4.00E+07	27153552	65.32%	47.92%	17.40%
HTG_TL_R1	Leaf	45°C	1.00E+08	91657981	89.85%	59.38%	30.47%
HTG_TR_R1	Root	45°C	5.00E+07	38269304	80.23%	58.53%	21.70%
HTG_TL_R2	Leaf	45°C	3.00E+07	29098139	86.09%	52.71%	33.38%
HTG_TR_R2	Root	45°C	4.00E+07	30888850	73.58%	56.09%	17.49%
HTG_TL_R3	Leaf	45°C	2.00E+07	19022430	78.49%	44.54%	33.95%
HTG_TR_R3	Root	45°C	3.00E+07	27025054	79.26%	53.28%	25.98%
HSG_CL_R1	Leaf	Control	4.00E+07	33176962	80.45%	56.37%	24.08%
HSG_CR_R1	Root	Control	9.00E+07	57597229	67.19%	48.00%	19.19%
HSG_CL_R2	Leaf	Control	4.00E+07	31974747	83.28%	47.34%	35.94%
HSG_CR_R2	Root	Control	5.00E+07	37151752	75.21%	49.93%	25.28%
HSG_CL_R3	Leaf	Control	4.00E+07	19861195	55.31%	30.62%	24.69%
HSG_CR_R3	Root	Control	3.00E+07	15390775	50.47%	30.65%	19.82%
HSG_TL_R1	Leaf	45°C	5.00E+07	20934726	44.41%	30.02%	14.39%
HSG_TR_R1	Root	45°C	3.00E+07	13284437	49.38%	35.12%	14.26%
HSG_TL_R2	Leaf	45°C	3.00E+07	25434882	87.18%	62.90%	24.28%
HSG_TR_R2	Root	45°C	4.00E+07	21105953	53.31%	38.69%	14.62%
HSG_TL_R3	Leaf	45°C	4.00E+07	34398084	84.12%	59.18%	24.94%
HSG_TR_R3	Root	45°C	4.00E+07	28901096	81.13%	58.38%	22.75%

HTG, heat-tolerant genotype; HSG, heat-sensitive genotype; CL, control condition of leaf tissue; CR, control condition of root tissue; TL, treatment condition of leaf tissue; TR, treatment condition of root tissue and R1, R2, and R3- biological replicates

### Identification of differentially expressed genes

DEGs were identified using the R bioconductor package edgeR version 3.42.4 (Robinson et al., 2010). TMM (Trimmed Mean of M-values) normalization method was applied to account for library size and composition differences across samples. The genes with the threshold of log2 fold change (FC) cutoff ≥2 and adjusted *p*-value ≤0.01 were selected as significant DEGs for further analyses and interpretations.

### Functional annotation of DEGs

To identify putative biological functions and pathways for the DEGs, the Gene Ontology (GO) and Kyoto Encyclopedia Of Genes And Genomes (KEGG) databases were searched for annotation using the Database for Annotation, Visualization and Integrated Discovery (DAVID, version 6.8) (https://david.ncifcrf.gov/summary.jsp) and SRplot (https://www.bioinformatics.com.cn) (Huang et al., 2007). This analysis provided all GO terms significantly enriched in DEGs compared to the genome background and filtered the DEGs corresponding to the biological functions. Significant GO and KEGG pathways were identified with the criterion of FDR-corrected *p*-value <0.05 (Yoav and Hochberg, 2000). Hyper-geometric statistical tests and Bonferroni correction methods were also applied (Sidak, 1967).

### Enrichment analysis of transcription factors

To identify enriched TF families functioning between genotypes under stressed and control conditions, a TF enrichment analysis was conducted. The identified DEGs were used as input and compared against the *Setaria italica* TF database from PlantRegMap (Tian et al., 2020). The DEGs were screened against 2,410 TFs, classified into 56 families, with a stringency *p*-value ≤0.01.

### Identifying the hub genes involved in the protein interaction network

Protein-Protein Interactions (PPI) are crucial to most biological processes, and understanding them is imperative for unravelling the molecular mechanisms underlying DEGs in transcriptomics. The DEGs obtained under heat stress were utilized to construct a network of PPIs. The STRING (Search Tool for the Retrieval of Interacting Genes and Proteins) database was employed and the required interaction score for the physical sub-network was set to default parameters to identify both validated and predicted protein-protein interactions to investigate protein functional relationships (http://string-db.org) (Szklarczyk et al., 2023). The resulting interactions were utilized to construct the PPIs network, which was then analyzed and visualized using Cytoscape v3.10.0 (https://cytoscape.org/) (Shannon et al., 2003). The gene network was examined using average path length to determine key global centrality parameters such as proximity and betweenness, centrality, and average degree. The PPIs network was designed to identify significant players or hub genes (nodes with the highest degree) involved in heat stress tolerance. The hub genes were identified and ranked based on degree using the Cytoscape plugin cytoHubba (Chin et al., 2014). The degree algorithm calculates the number of direct interactions of each gene in the PPI network. Hub genes were recognized based on the higher number of connections or degrees over other genes.

## Results

### Genome-wide transcriptome data statistics

Genome-wide transcriptome profiling was conducted in leaf and root tissues of HTG and HSG under control and heat-treated conditions to examine the transcriptome regulation of tissue-specific genes in response to heat stress. We extracted a total of one billion raw reads using Illumina sequencing technology, of which 760 million reads, after rigorous quality testing and data cleaning, were mapped to the pearl millet reference genome from established 24 RNA-Seq cDNA libraries. Over 72.43% of the high-quality reads were effectively mapped to the pearl millet reference genome, of which 48% were uniquely mapped, whereas 23% illustrated multiple genomics locations (**Table 1**).

### Gene expression profile analysis of HTG and HSG under heat stress

**Figure 1:**
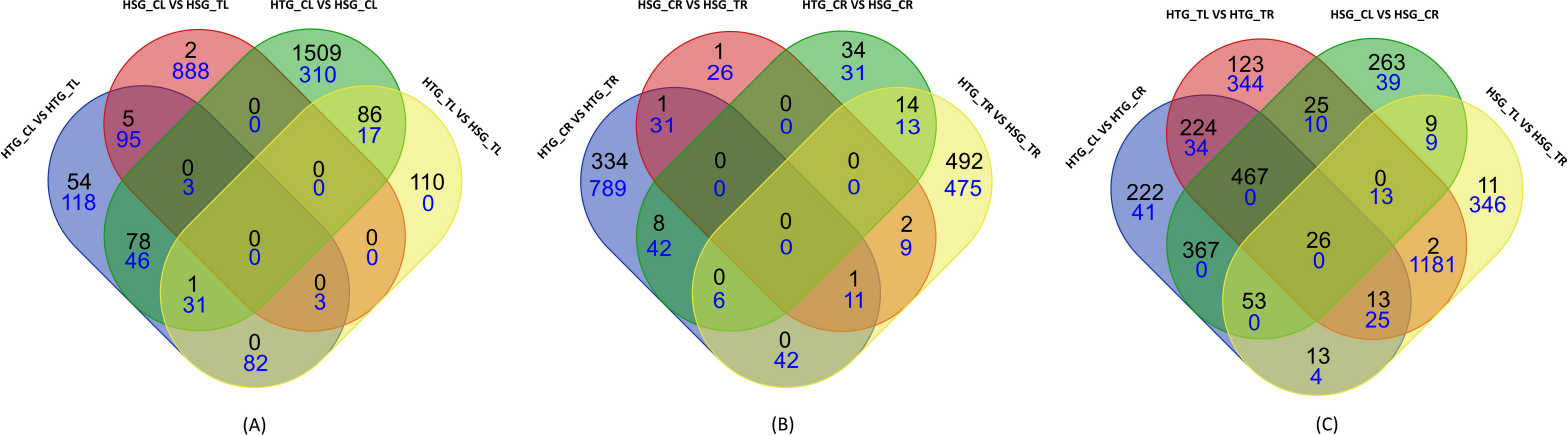
Up- (black) and down-regulated (blue) DEGs across comparisons in **(A)** leaf, **(B)** root, and **(C)** leaf vs root under heat stress treatments.

The expressed genes were generated and discovered using a stringent log2 FC ≥2 threshold and *p-*value <0.01 to comprehend their biological function better. **Table 2** presents statistics of the DEGs between the twelve possible combinations. A thorough examination of tissue-specific comparisons between control and stress conditions in both genotypes produced 13,464 DEGs, of which 6,932 showed down-regulation and 6,532 showed up-regulation. In the leaf category, it was found that 1,665 genes were down-regulated, and 914 genes were up-regulated out of a total of 2,579 genes identified. Comparatively, 2,016 genes were induced in the root category, while 1,907 genes were suppressed among the 3,923 genes examined. The comparison between leaf and root tissues revealed that the most DEGs, with 3,360 showing lower expression and 3,602 displaying higher expression, out of 6,962 genes studied. In addition, tissue-specific evaluation of HTG and HSG revealed that 489 and 15 genes were commonly up-regulated and down-regulated, respectively (**Figure 1**).

**Table 2:** Pairwise comparison of differentially expressed genes (DEGs) in heat-tolerant and heat-sensitive genotypes under different heat stress conditions.

S. No	Combinations	Total DEGs	Down-regulated DEGs	Up-regulated DEGs
1	HTG_CL *vs* HTG_TL	1265	921	344
2	HSG_CL *vs* HSG_TL	82	77	5
3	HTG_CL *vs* HSG_CL	148	92	56
4	HTG_TL *vs* HSG_TL	1084	575	509
5	HTG_CR *vs* HTG_TR	516	378	138
6	HSG_CR *vs* HSG_TR	996	989	7
7	HTG_CR *vs* HSG_CR	2081	407	1674
8	HTG_TR *vs* HSG_TR	330	133	197
9	HTG_CL *vs* HTG_CR	231	104	127
10	HTG_TL *vs* HTG_TR	2817	1607	1210
11	HSG_CL *vs* HSG_CR	951	71	880
12	HSG_TL *vs* HSG_TR	2963	1578	1385

HTG, heat-tolerant genotype; HSG, heat-sensitive genotype; CL, control condition of leaf tissue; CR, control condition of root tissue; TL, treatment condition of leaf tissue; TR, treatment condition of root tissue.

### Identification of heat-responsive DEGs in leaf

The leaf transcriptome analysis identified 2,579 expressed transcripts, with 1,665 showing down-regulation and 914 showing up-regulation (**Table 2**). The Venn diagram (**Figure 1**) represents the total DEGs identified in the leaf tissues of both genotypes under control and treated conditions. Significant expression patterns were observed in various comparisons. For instance, in HTG_CL vs HSG_CL, 92 genes were down-regulated, and 56 genes were up-regulated. Similarly, in HTG_CL vs HTG_TL, we found 921 suppressed and 344 induced genes. Moreover, in HSG_CL vs HSG_TL, 77 genes showed lower expression while only five genes were over-expressed, and in HTG_TL vs HSG_TL, 575 genes were down-regulated while 509 were up-regulated.

The comparisons indicate a trend where genes responsible for various mechanisms and pathways in pearl millet leaves tend to experience suppression under stress conditions. The sensitive genotype showed a significant increase in down-regulated DEGs, suggesting its heightened vulnerability to heat stress. This implies that the HSG genotype is more sensitive to the negative impacts of high temperatures than the HTG genotype.

On comparing the transcript abundance profile among all four combinations, we identified that *HATPase domain-containing protein, SHSP, protein kinases, lipoxygenase, chlorophyll a-b binding protein*, and *BHLH* were significantly up-regulated. In contrast, *stachyose synthase, BURP, lipase_3, lipase_GDSL domain-containing protein, cytochrome C* and *b559, BZIP, dirigent protein*, and *lipoxygenase* showed significant down-regulation. Four of the top 10 highest up-regulated genes were expressed in HTG_TL *vs* HSG_TL, and of the top 10 down-regulated genes, HSG_CL *vs* HSG_TL included five highly-expressed genes. The significant up-regulation and down-regulation of expressed genes in the leaf are summarized in **Table 2**. Among all the genes expressed, lipase was expressed in all the combinations except HSG_CL *vs* HSG_TL, which was down-regulated (-11.5 FC) in the sensitive genotype. The comparison between tolerant and sensitive genotypes under control conditions revealed down-regulation of *Receptor-like serine/threonine-protein kinase*, *AP2/ERF domain-containing protein*, and *ABC transporter*. In contrast, there was a significant up-regulation, ranging from -11 to 14-fold, of *Lipase_3 domain-containing protein, NAD(P)H dehydrogenase*, and *Laccase*. P*eroxidase*, *jmjC protein* (chromatin remodeling and histone modification), *WRKY*, *24.1kDa HSP, LEA protein* (prevents protein aggregation under stress), *chitinase, elongation factor, NAC*, and *DNA helicase* were induced several folds. On the other hand, *NAD(P)H dehydrogenase, AAI domain-containing protein, bidirectional sugar transporter SWEET, FE2OG dioxygenase, phytocyanin*, and *ABC transporter* genes were down-regulated across most of the combinations.

*Chlorophyll a-b binding* protein involved in photosynthetic activity was highly up-regulated in the tolerant genotype under treatment conditions. *Peroxidase* and *thioredoxin* enzymes belonging to the hydrogen peroxide catabolic process were more repressed in HSG. The *lipoxygenase* that plays a role in fatty acid biosynthesis and lipid oxidation was down-regulated across all combinations except HTG_TL *vs* HSG_TL, where it was five-fold induced. Genes involved in glutathione metabolic processes, such as *glutathione synthetase* and *glutathione transferase*, were mostly repressed in the treated sensitive genotype. *Cytochrome b599* and *photosystem I* and *II* are cellular components of the chloroplast thylakoid membrane and were mostly down-regulated in the stressed HSG. In contrast, these genes displayed less suppression in the tolerant genotype than in the sensitive genotype. Extracellular region enzymes *expansin*, involved in cell wall organization, was up-regulated threefold in HTG_TL *vs* HSG_TL. In contrast, *L-ascorbate oxidase* was down-regulated threefold in HTG_CL *vs* HTG_TL. *Bidirectional sugar transporter SWEET* was down-regulated several folds in the HTG_TL *vs* HSG_TL and up-regulated in the HTG_CL *vs* HTG_TL. *Amino acid permease* involved in the transmembrane transporter activity of amino acid, *alpha-amylase* and *PsbP* domain-containing protein, which is part of PSII, was more down-regulated in the sensitive genotype than in the tolerant genotype under heat stress.

### Identification of heat-responsive DEGs in root

Transcriptome analysis of all comparisons of root samples identified 3923 genes, of which 2016 and 1907 genes were induced and repressed, respectively (**Figure 1**). The Venn diagram represents commonly up-regulated and down-regulated genes under control and stress conditions in the roots of HTG and HSG. While analyzing the expression dynamics, on comparing HTG control with treatment, we found that 138 genes were up-regulated, and 378 genes were down-regulated. We observed more down-regulated genes (989) and fewer induced genes (Ashraf and Hafeez, 2004) in the control *vs* treatment of sensitive genotype. Comparing the control conditions of both genotypes, it was observed that 1674 genes were up-regulated while 407 genes were down-regulated. When comparing the treatment conditions of both genotypes, 197 genes showed over-expression, while 133 transcripts showed under-expression (**Table 2**).

In contrast to the leaf samples, the root tissues showed a higher number of up-regulated DEGs (2016). This up-regulation of genes in the roots under heat stress conditions suggests a different molecular response than the leaves. The HSG exhibited a notable number of down-regulated DEGs in the roots, indicating a suppression of gene expression, specifically in this genotype under heat stress.

From the present study, we identified DEGs encoding *peroxidase, protein kinase* and *AAI domain-containing proteins* that were significantly expressed across all combinations. *Aldehyde oxygenase, involved in lipid biosynthesis, bidirectional sugar transporter SWEET*, and *Fe2OG dioxygenase* domain-containing protein, was up-regulated in the HTG combinations, whereas in the sensitive genotype, these genes were down-regulated.

In the sensitive genotypes treatment condition, it was revealed that *17.9kDa HSP, chlorophyll a-b binding protein, PSII, RuBisCO, thioredoxin, malate dehydrogenase, potassium transporter, ABC transporter, copper transporter, glutamine synthetase, glutathione transferase, nitrate reductase, PsbP protein, Clp protease, temperature-induced lipocalin (TIL)* that protects chloroplasts from ion toxicity, *PSI, phosphate transporter, MAPK, MYB, RING-type E3 ubiquitin transferase* and *zinc finger proteins* were down-regulated several folds. Enzymes *lipase* and *lipoxygenase, laccase* and *stachyose synthase*, and the *fructose bisphosphate aldolase* that participates in carbohydrate degradation in the glycolysis cycle were primarily down-regulated in the sensitive genotypes. Under control conditions, comparison between genotypes revealed suppressed expression of *Glutathione S-transferase, Protein kinase*, and *Sucrose synthase*. Conversely, there was up-regulation of *terpene synthase*, which is involved in the synthesis of secondary metabolites, as well as *Phosphoenolpyruvate carboxykinase* and *Fructose-bisphosphate aldolase*, both of which are involved in carbohydrate biosynthesis. In the treatment comparison of both the genotypes, *stromal 70kDa HSP* was down-regulated, in contrast to *calcium-binding protein 60*, which was induced two-fold. *Xyloglucan endotransglucosylase/hydrolase* was down-regulated across all the combinations, but the level of expression was found more suppressed (-6 folds) in the sensitive genotype in comparison to the tolerant. The results from the present study align with the comparative transcriptomic research conducted on *Agrostis species*, which revealed the activation of root antioxidant enzymes, genes involved in respiration, HSPs, and transcription factors aided tolerant genotype to adapt better to heat stress by maintaining growth and development (Huang, Bingru et al., 2012).

### Comparison of heat-responsive DEGs in leaf *vs* root

Leaf and root stress treatments were compared to identify the tissue-specific expression of genes involved in transcriptional regulation of both pearl millet genotype’s responses to heat stress. The comparative transcriptomic analysis between leaf and root tissues revealed 6,962 expressed genes, with 3,602 showing up-regulation and 3,360 displaying down-regulation (**Figure 1**). Under control conditions, the comparison indicated 127 up-regulated genes and 104 down-regulated in the tolerant genotype, whereas 880 genes were induced and 71 genes were suppressed in the sensitive genotype. Under heat stress, HTG exhibited 1,210 up-regulated genes, while 1607 genes were down-regulated in the root. Meanwhile, 1385 genes were up-regulated, and 1,578 genes were down-regulated in the HSG (**Table 2**). The analysis of expression dynamics demonstrated distinct transcriptional responses to heat stress in the different tissues of both genotypes, highlighting their divergent molecular mechanisms in coping with environmental challenges.

*AP2/ERF, auxin response factor, WRKY, NAC, lipoxygenase, lipase-GDSL, calmodulin-binding protein, PIP26, PIP11, respiratory burst oxidase, ring-type E3 ubiquitin transferase, sucrose synthase, terpene synthase, patatin, cytokinin dehydrogenase, bidirectional sugar transporter SWEET, and calcium uniporter* showed induced expression across all the comparisons except in control condition of the tolerant genotype. In comparing leaf and root tissues under control conditions, the tolerant genotype showed enhanced expression of *dirigent protein* and *Photosystem I P700*. Conversely, in the sensitive genotype, *protein kinase*, *peroxidas*e, and *the bidirectional sugar transporter SWEET* were significantly down-regulated. Some of the genes *Burp, laccase, lipase, BZIP, potassium transporter, protein kinase, peroxidase, and glutathione transferase* displayed distinct expression patterns across all combinations of HTG under control and stress conditions. Notably*, FAD-binding protein, xyloglucan endotransglucosylase/hydrolase*, and *BHLH* expression were positively regulated, indicating they were significantly more active in tolerant genotype’s roots.

Genes such as *PEPC, chlorophyll a-b binding protein, cytochrome p450, fructose-bisphosphate aldolase, B-box zinc finger, ferredoxin–NADP reductase, PsbP, PSI, PSII, RNA helicase, Ring-CH-type domain-containing protein, MYB, sHsp17.0A, superoxide dismutase, stromal HSP70, thioredoxin, HSF protein, glutathione peroxidase, copper transporter*, and *CP12 domain-containing protein* conversely demonstrated suppression of transcription and notable decrease in the expression levels. Furthermore, *zeaxanthin epoxidase, zinc finger protein, RAP domain-containing protein, HATPase, GrpE protein, elongation factor*, and *catalase* expression were down-regulated several folds in the root tissues.

### Functional annotation and pathways enrichment of expressed genes

GO enrichment analysis was conducted to identify and describe putative DEGs between tolerant and sensitive genotypes under heat stress. A complete set of all DEGs was aligned against the DAVID GO database, resulting in the classification of annotated genes to three fundamental GO components: cellular component (CC) and molecular functions (MF) biological process (BP). Annotated genes included 452 for 19 CC, 331 for 22 MF, and 251 for 36 BP. The most substantially over-represented GO terms for the up-regulated genes were chloroplast thylakoid membrane, heme binding, and hydrogen peroxide catabolic process in the CC, MF, and BP categories, respectively. Similarly, for down-regulated genes, chloroplast, peroxidase activity, and hydrogen peroxide catabolic process were prevalent in each component.

The GO classification of expressed genes in different tissue-specific categories for both genotypes is represented in **Supplementary Figure S1**. In the leaf, out of 2552 genes analyzed, 1259 were sorted into three functional categories: 610 in BP, accounting for 48.5%, 644 in CC, making up 51.2%, and 870 in MF, representing 69.1%. For root tissues, the analysis revealed that out of 3885 genes studied, 1628 were classified as follows: 777 (47.7%) in BP, 858 (52.7%) in CC, and 1100 (67.6%) in MF. While comparing leaf versus root categories with a total of 6879 genes, 2569 were distributed across three classes: 1218 (47.4%) in BP, 1361 (53%) in CC, and 1777 (69.2%) in MF.

**Figure 2:**
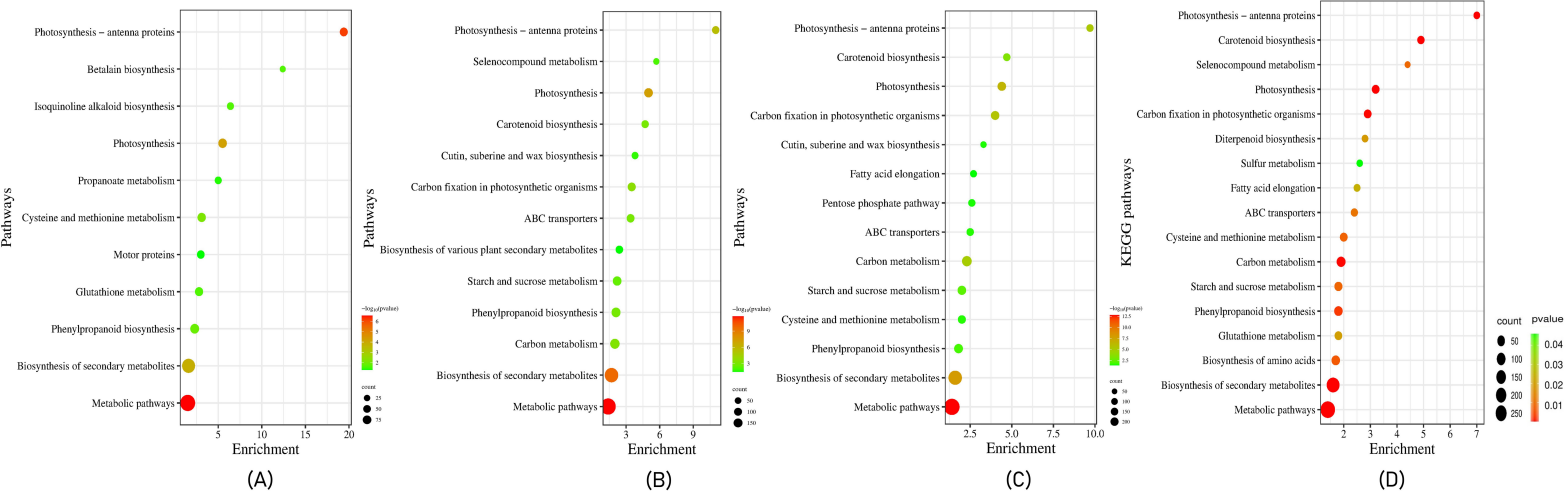
Biological pathways enrichment of the DEGs in **(A)** the leaves and roots of tolerant *vs* sensitive genotypes under heat stress conditions. **(B)** tolerant genotype leaf *vs* root **(C)** sensitive genotypes leaf *vs* root and **(D)** top 17 biological pathways enrichment of the DEGs in the leaves and roots of pearl millet genotypes under heat stress conditions. The size of the dot represents the number of the DEGs involved in each pathway. The color of the dot represents the p-value of each pathway; the pathways with *p* value ≤ 0.05 were significantly enriched pathways.

In our study, KEGG analysis was used to assign biological pathways to the identified expressed genes. The comparative study of the tolerant and sensitive genotypes under both control and stressed conditions suggested a significant enrichment of genes that regulate metabolic and biosynthesis of secondary metabolites pathways. Under control conditions, the protein modification pathway in leaves and the carotenoid biosynthesis pathway in roots were enriched in the tolerant compared to the sensitive genotype. Under stressed conditions, tissue-specific differences in pathway enrichment were observed. In leaf tissues of the tolerant genotype, genes associated with photosynthesis, cysteine and methionine metabolism, and phenylpropanoid biosynthesis pathways were significantly enriched, whereas, in roots, the isoquinoline alkaloid biosynthesis pathway was enriched. When comparing control and stressed conditions within the tolerant genotype, genes involved in photosynthesis, linoleic acid metabolism, and carotenoid biosynthesis pathways were expressed in leaf tissues, while in roots, genes involved in the biosynthesis of amino acids and starch and sucrose metabolism pathways were expressed (**Figure 2**). The pathway enrichment analysis revealed that 1,172 of the expressed genes in the tolerant genotype were associated with 17 significant pathways. Additionally, metabolic pathways, biosynthesis of secondary metabolites, and photosynthetic pathways were highly enriched in both overexpressed and underexpressed genes (**Figure 2**). These findings provide valuable insights into the specific functions, processes, and pathways associated with the heat stress response in pearl millet.

We successfully annotated a subset of the DEGs, revealing valuable information about their molecular functions, biological processes, and cellular components. The analysis also highlighted the presence of significant number of 1906 uncharacterized proteins, indicating potential novel genes associated with the heat stress response in pearl millet. A considerable proportion of the 60 transcripts remained unannotated, highlighting the need for future investigations to unravel their roles and functions. The identification of a substantial number of DEGs across various comparisons underscores the significant impact of heat stress on gene expression. It highlights the intricate molecular mechanisms underlying the plant’s response to heat stress.

### Enrichment analysis of TFs under heat stress

To better understand the molecular mechanisms underlying the response to heat stress in pearl millet, TF enrichment analysis was performed on DEGs from HTG and HSG under both control and stressed conditions. This analysis revealed several TF families with significant enrichment, suggesting their potential regulatory roles under heat stress. The top five enriched TFs and their associated GO terms from each comparison category are summarized in **Table 3**. The comparisons included HTG *vs* HSG under both control and stressed conditions, as well as HTG and HSG stressed *vs* control conditions.

**Table 3:** Enriched transcription factors in tolerant and sensitive genotypes under control and stress conditions.

Category	TF	Gene Input	Target Genes	p-value	q-value(BH)	Gene ontology
**HTG *vs* HSG control**	CPP	649	57	6.84E-04	1.65E-01	GO:0009934 regulation of meristem structural organization GO:0048444 floral organ morphogenesis GO:0051302 regulation of cell division GO:0005634 nucleus GO:0016021 integral component of membrane
	bZIP	649	27	1.85E-03	2.23E-01	GO:0045893 positive regulation of transcription, DNA-templated GO:0005634 nucleus GO:0003700 transcription factor activity, sequence-specific DNA binding GO:0043565 sequence-specific DNA binding
	MYB	649	20	3.00E-03	2.39E-01	GO:0006355 regulation of transcription, DNA-templated GO:0005634 nucleus GO:0003677 DNA binding
	GRAS	649	146	4.82E-03	2.39E-01	GO:0006355 regulation of transcription, DNA-templated GO:0009723 response to ethylene GO:0009737 response to abscisic acid GO:0009867 jasmonic acid mediated signaling pathway GO:0009938 negative regulation of gibberellic acid mediated signaling pathway GO:0010187 negative regulation of seed germination GO:0010218 response to far red light GO:0042176 regulation of protein catabolic process GO:0042538 hyperosmotic salinity response GO:2000033 regulation of seed dormancy process GO:2000377 regulation of reactive oxygen species metabolic process GO:0005634 nucleus GO:0003700 transcription factor activity, sequence-specific DNA binding GO:0044212 transcription regulatory region DNA binding
	bHLH	649	15	4.97E-03	2.39E-01	GO:0009637 response to blue light GO:0005634 nucleus GO:0046983 protein dimerization activity
**HTG *vs* HSG stressed**	bZIP	363	18	2.93E-03	4.69E-01	GO:0006355 regulation of transcription, DNA-templated GO:0009737 response to abscisic acid GO:0005829 cytosol GO:0003700 transcription factor activity, sequence-specific DNA binding GO:0043565 sequence-specific DNA binding GO:0044212 transcription regulatory region DNA binding
	bZIP	363	16	6.23E-03	4.69E-01	GO:0045893 positive regulation of transcription, DNA-templated GO:0005634 nucleus GO:0003700 transcription factor activity, sequence-specific DNA binding GO:0043565 sequence-specific DNA binding
	MYB	363	15	1.13E-02	4.69E-01	GO:0003677 DNA binding
	G2-like	363	14	2.77E-02	4.81E-01	GO:0006355 regulation of transcription, DNA-templated GO:0005634 nucleus GO:0003677 DNA binding
	bZIP	363	14	3.41E-02	5.07E-01	GO:0009409 response to cold GO:0009414 response to water deprivation GO:0009651 response to salt stress GO:0009737 response to abscisic acid GO:0009739 response to gibberellin GO:0010152 pollen maturation GO:0010187 negative regulation of seed germination GO:0010200 response to chitin GO:0045893 positive regulation of transcription, DNA-templated GO:0048316 seed development GO:0005634 nucleus GO:0003700 transcription factor activity, sequence-specific DNA binding GO:0043565 sequence-specific DNA binding
**HTG stressed *vs* control**	G2-like	483	25	3.52E-03	4.44E-01	GO:0006355 regulation of transcription, DNA-templated GO:0005634 nucleus GO:0003677 DNA binding
	bZIP	483	12	4.57E-03	4.44E-01	GO:0006355 regulation of transcription, DNA-templated GO:0003700 transcription factor activity, sequence-specific DNA binding GO:0043565 sequence-specific DNA binding
	NAC	483	15	5.84E-03	4.44E-01	GO:0006355 regulation of transcription, DNA-templated GO:0009753 response to jasmonic acid GO:0045995 regulation of embryonic development GO:0048317 seed morphogenesis GO:0080060 integument development GO:0005634 nucleus GO:0044212 transcription regulatory region DNA binding
	NAC	483	10	7.62E-03	4.44E-01	GO:0006355 regulation of transcription, DNA-templated GO:0010072 primary shoot apical meristem specification GO:0010160 formation of organ boundary GO:0010223 secondary shoot formation GO:0048366 leaf development GO:0048504 regulation of timing of organ formation GO:0005634 nucleus GO:0003677 DNA binding
	bHLH	483	11	1.32E-02	4.61E-01	GO:0009637 response to blue light GO:0005634 nucleus GO:0046983 protein dimerization activity
**HSG stressed *vs* control**	bZIP	338	9	8.06E-04	1.42E-01	GO:0006355 regulation of transcription, DNA-templated GO:0007231 osmosensory signaling pathway GO:0008272 sulfate transport GO:0009294 DNA mediated transformation GO:0009652 thigmotropism GO:0009970 cellular response to sulfate starvation GO:0045596 negative regulation of cell differentiation GO:0051170 nuclear import GO:0005634 nucleus GO:0005829 cytosol GO:0003682 chromatin binding GO:0003700 transcription factor activity, sequence-specific DNA binding GO:0043565 sequence-specific DNA binding GO:0043621 protein self-association GO:0051019 mitogen-activated protein kinase binding
	ARF	338	5	1.67E-03	1.42E-01	GO:0006355 regulation of transcription, DNA-templated GO:0009734 auxin-activated signaling pathway GO:0009908 flower development GO:0005634 nucleus GO:0003677 DNA binding GO:0005515 protein binding
	CPP	338	32	2.33E-03	1.42E-01	GO:0009934 regulation of meristem structural organization GO:0048444 floral organ morphogenesis GO:0051302 regulation of cell division GO:0005634 nucleus GO:0016021 integral component of membrane
	GRAS	338	83	2.41E-03	1.42E-01	GO:0006355 regulation of transcription, DNA-templated GO:0009723 response to ethylene GO:0009737 response to abscisic acid GO:0009867 jasmonic acid mediated signaling pathway GO:0009938 negative regulation of gibberellic acid mediated signaling pathway GO:0010187 negative regulation of seed germination GO:0010218 response to far red light GO:0042176 regulation of protein catabolic process GO:0042538 hyperosmotic salinity response GO:2000033 regulation of seed dormancy process GO:2000377 regulation of reactive oxygen species metabolic process GO:0005634 nucleus GO:0003700 transcription factor activity, sequence-specific DNA binding GO:0044212 transcription regulatory region DNA binding
	bZIP	338	16	3.08E-03	1.46E-01	GO:0045893 positive regulation of transcription, DNA-templated GO:0005634 nucleus GO:0003700 transcription factor activity, sequence-specific DNA binding GO:0043565 sequence-specific DNA binding

CPP, cystein-rich polycomb-like protein; bZIP, Basic leucine zipper; MYB, myeloblastosis viral oncogene homolog; G2-like, Golden2-Like; NAC, NAM, ATAF, and CUC; bHLH, basic/helix-loop-helix; ARF, Auxin response factors; and GRAS, Gibberellic acid; Repressor of GA and Scarecrow).

The significantly enriched TF families included *bZIP, MYB, bHLH, G2-like*, and *NAC*. The *bZIP* family was prominently enriched across all comparisons. In the tolerant *vs* sensitive genotype under stress, the *bZIP* family had 48 target genes, with GO terms indicating its involvement in responses to abiotic stresses. Additionally, the G2-like family, with 39 target genes, along with *MYB* and *NAC* families, with 15 and 25 target genes respectively, were significantly enriched and predominantly found in the tolerant genotype under heat stress. These TFs are involved in various biological processes and molecular functions related to transcription regulation, abiotic stress responses, and developmental processes. These findings suggest the crucial role of these TF families in gene regulation under heat stress in pearl millet, offering valuable targets for further functional studies to elucidate their roles in stress response and plant development.

### Network analysis of core heat stress-responsive genes: hub genes, functional enrichment, and molecular insights

The PPI network of core heat stress-responsive genes comprised 260 nodes and 3336 edges, forming two distinct clusters− one large and one small. The genes in the larger clusters were most significantly enriched in functions related to ribosomes, metabolic pathways, carbon metabolism and biosynthesis of secondary metabolites. The genes in smaller clusters were predominantly enriched in starch and sucrose metabolism along with metabolic pathways.

Key hub genes exhibiting high connectivity (degree >70) included *KOW domain-containing proteins*, various *ribosomal proteins* (such as *uS12, bL36*, and *bL17*), *S5 DRBM domain-containing proteins, elongation factor Tu (EF Tu)*, and several uncharacterized proteins. These hub genes interacted with other crucial proteins, such as *BAG domain-containing protein, Superoxide dismutase, PsbP domain-containing protein, HATPase_c, WRKY, ERF, Fes1 domain-containing protein, Pyruvate kinase, Acyl carrier protein, chlorophyll a-b binding protein, Thioredoxin domain-containing protein*, and *EF Ts*.

**Figure 3:**
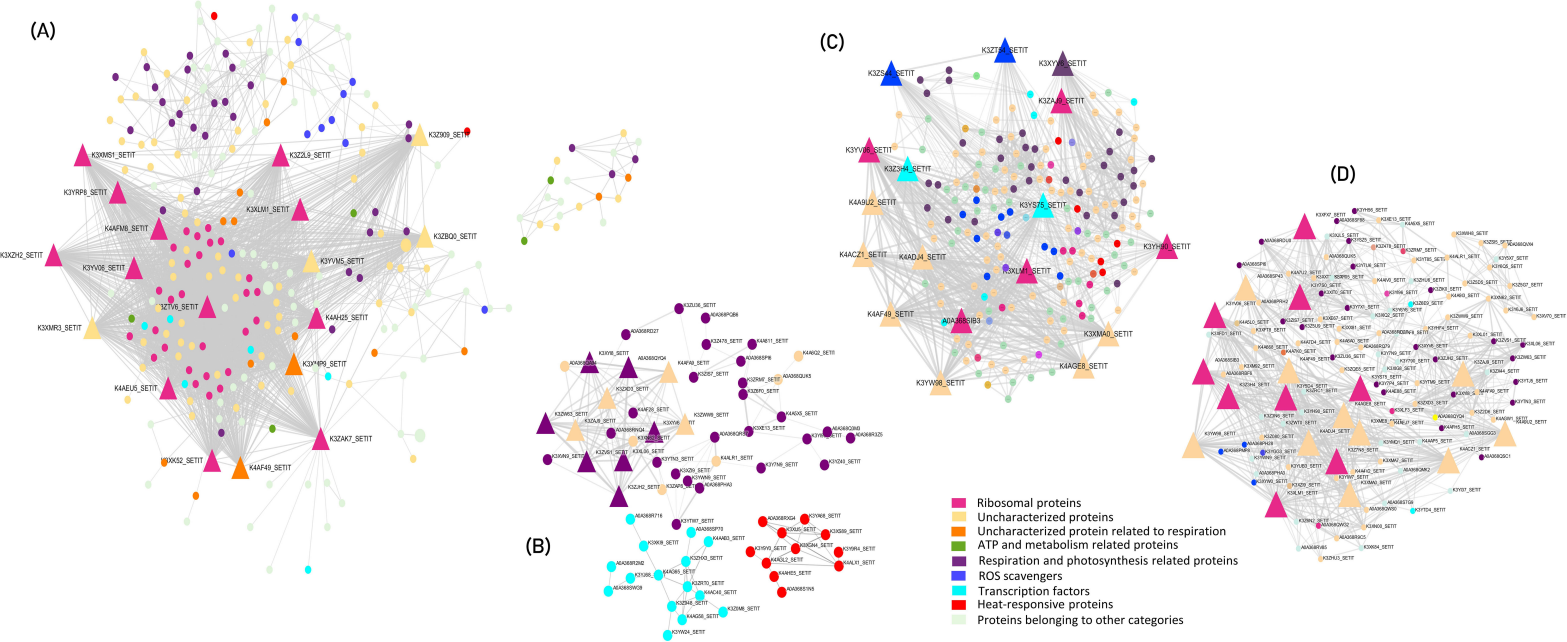
Protein-Protein Interaction network of **(A)** overall genes expressed in response to heat stress **(B)** DEGs in leaf and root of tolerant *vs* sensitive genotype **(C)** DEGs in the leaf *vs* root of tolerant genotype and **(D)** DEGs in the leaf *vs* root of sensitive genotype. Triangular nodes represent the hub genes identified and circular nodes represent the crucial genes involved in heat stress responsive mechanisms.

In this study, one notable hub gene, namely the *KOW domain-containing protein*, is a nuclear RNA binding protein essential for plants’ innate immunity against various biotic and abiotic stresses (Aksaas et al., 2011). We observed 39 genes related to ribosomal proteins, including S5 DRBM domain-containing protein, exhibited high connectivity ranging from 32 to 91 degrees (**Figure 3**). The results indicated that heat stress caused detrimental effects on the expression of ribosomal proteins of the large subunit genes due to their decreased stability. Ribosomal proteins are involved in the selective synthesis of important proteins in response to heat stress. These ribosomal proteins modulate protein accumulation under stress conditions and their regulation reduces energy consumption.

Another significant hub gene, *EF Tu* with a degree of 71, is a highly conserved GTP-binding protein essential for translation in many species, including prokaryotes and eukaryotes (Xifeng et al., 2018). Studies in spring wheat showed its accumulation in response to heat stress, with higher levels correlating with improved heat tolerance (Bukovnik et al., 2009). Notably, the recombinant maize pre-EF-Tu was stable at 45°C and acted as a molecular chaperone, reserving protein stability under heat stress by preventing thermal protein aggregation (Rao et al., 2004).

Genes associated with *HATPase*, photosynthesis, carbon metabolism and TFs including *WRKY* and *ERF* interacted with the hub genes. *BAG domain-containg protein*, recruited molecular chaperones using their domains under stress conditions to target proteins and changed their function by altering the protein conformation (Ding et al., 2020). BAG proteins regulate various physiological processes such as apoptosis, tumor induction, stress response and cell cycle. BAGs also regulate HSP chaperone proteins (positively or negatively) and form complexes with various transcription factors. At the transcriptional level, BAG family genes in plants have key roles in the PCD processes which range from growth, and tolerance to fungi to abiotic stress tolerance.

Comparative analysis of tolerant *vs* sensitive genotypes under heat stress conditions revealed a primary cluster and two sub-clusters, where hub genes in the primary cluster (degree >10) were predominantly associated with respiration and photosynthetic pathways. The two sub-clusters were enriched for transcription factors, such as NAC, and stress-responsive proteins, including small heat shock proteins (sHSPs). In the leaf *vs* root comparison of the tolerant genotype, hub genes identified were elongation factor Tu (EF Tu) with a degree of 53 and several uncharacterized ribosomal proteins. Conversely, in the sensitive genotype, hub genes comprised S5 DRBM domain-containing proteins, EF Tu, and other uncharacterized proteins, with degrees ranging from 20 to 42 (**Figure 3**). PPI network highlighted the intricate interplay among various proteins involved in the heat-stress response and explained potential mechanisms underlying heat tolerance in pearl millet.

### Unveiling the evolutionary significance of heat shock proteins and transcription factors across related crop species under heat stress

Our transcriptome results highlighted the crucial involvement of HSPs and TFs in the heat stress response of pearl millet. Therefore, 15 HSP and 179 TF-related genes identified in pearl millet were searched against rice, maize, proso millet, sorghum and foxtail millet genomes using BLAST for the identification of orthologous genes. Foxtail millet showed the maximum gene homology, sharing 11 HSP-related genes with pearl millet, followed by proso millet, rice, maize, and sorghum (Chandel et al., 2013; Nagaraju et al., 2015; Singh et al., 2016; Li and Howell, 2021; Barthakur and Bharadwaj, 2022). TF-related genes also showed maximum homology, with foxtail millet sharing 137 genes, followed by proso millet, sorghum, rice and maize (Chandel et al., 2013; Nagaraju et al., 2015; Singh et al., 2016; Li and Howell, 2021; Barthakur and Bharadwaj, 2022). Annotations were assigned to the identified orthologues to understand their functionality in the respective species.

**Figure 4:**
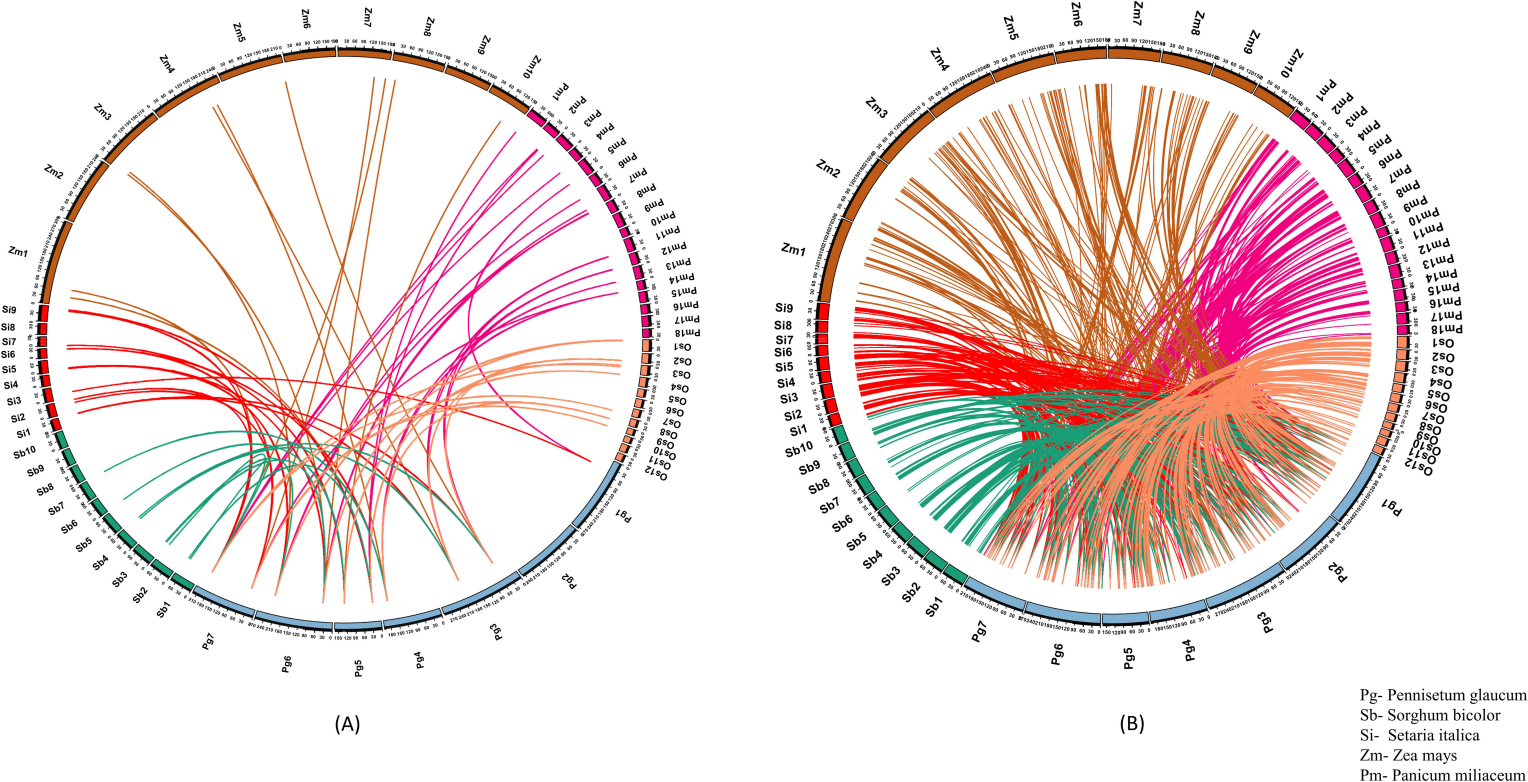
Comparative synteny plot demonstrating the orthologous genes related to **(A)** HSPs and **(B)** TFs in pearl millet to rice, maize, proso millet, sorghum and foxtail millet. The numeric values in the plot represent the chromosome number.

Our analysis revealed that several genes had more than one orthologous sequence across different crops. Specifically, in proso millet, out of the total of 132 identified genes associated with TF, 109 genes had more than one ortholog sequence. Similarly, out of the 10 identified genes related to HSP, 7 genes had more than one ortholog sequence. (**Figure 4**). All crops except sorghum had 2 genes associated with HSP, while sorghum had only one gene with more than one ortholog. Rice, sorghum, foxtail millet, and maize had 48, 43, 45, and 58 TFs, respectively, had more than one ortholog sequence.

Foxtail millet showed the highest similarity to HSPs and TFs by capturing 13 and 184 ortholog sequences from 15 HSPs and 179 TFs, respectively (**Figure 4**). In proso millet, 18 ortholog sequences were identified for 10 HSPs. Out of these, 13 sequences showed more than 85% similarity and three sequences showed more than 70% similarity to pearl millet HSPs. Similarly, 285 ortholog sequences were identified for 132 TFs, of which 91 sequences showed more than 85% similarity and 81 sequences showed more than 70% similarity to pearl millet TFs.

In the study, it was found that Sorghum had nine ortholog sequences for eight HSPs. Out of these, six sequences showed more than 85% similarity and three sequences showed more than 70% similarity. On the other hand, 172 ortholog sequences were identified for TFs, of which 32 sequences showed more than 85% similarity and 44 sequences showed more than 70% similarity (**Figure 4**). The comparison between pearl millet and maize resulted in the identification of 11 ortholog sequences, which were mapped to nine genes related to HSP. Additionally, 205 ortholog sequences were detected for 125 genes related to TF. Our research explained the conservation and diversity of HSPs and TFs engaged in the response to heat stress among different crop species.

## Discussion

**Figure 5:**
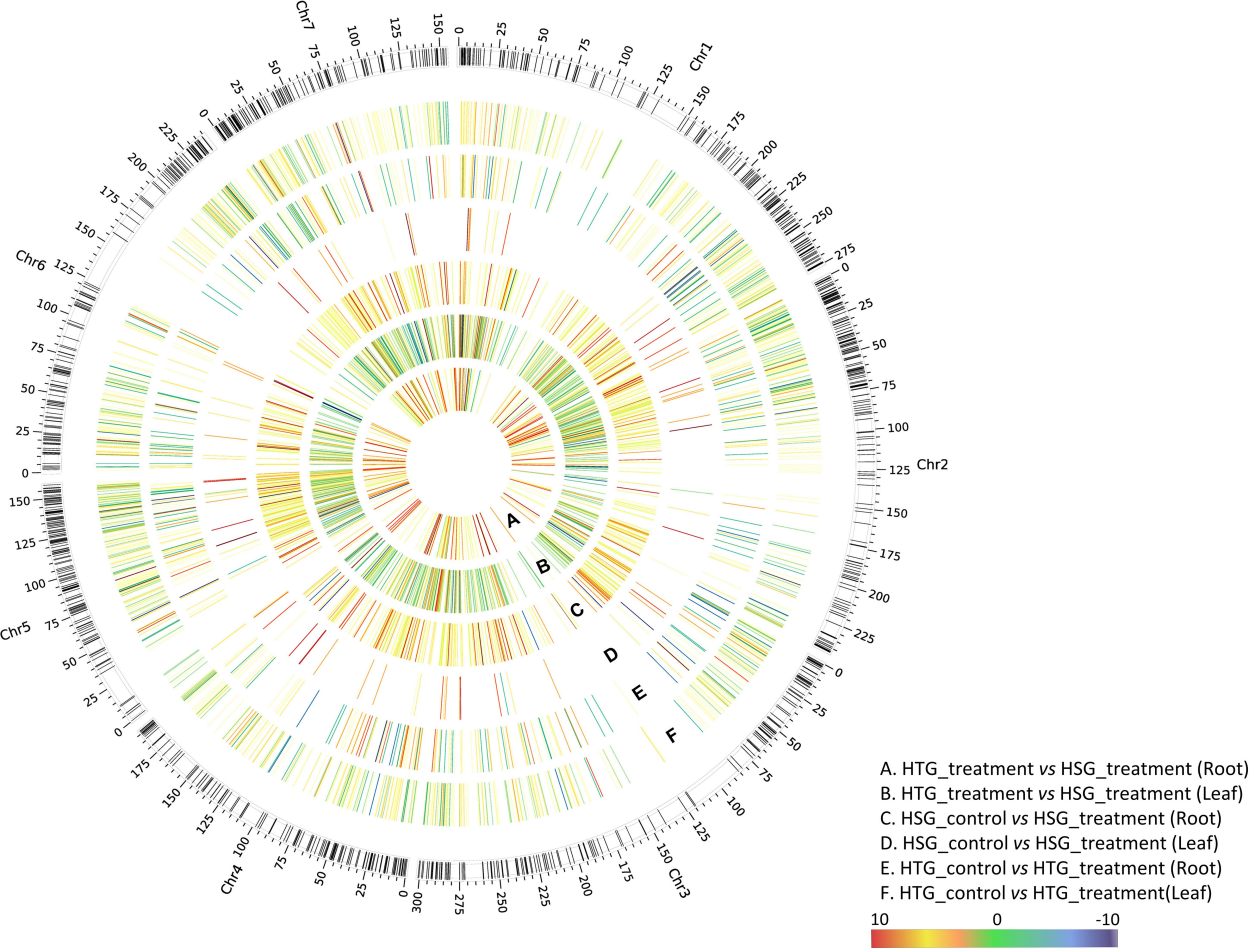
Circos plot represents differential expression pattern of heat-responsive genes in the HTG and HSG under control and treatment conditions. The outermost ring shows pearl millet chromosomes. The six rings namely, **(A–F)**, explain the expression pattern of genes across different pairwise comparisons of HTG and HSG.

Our results provide an understanding of the transcriptional responses of pearl millet to heat stress in leaf and root tissues. We discovered a total of 13,464 DEGs across all comparisons using comprehensive analysis, revealing the considerable influence of heat stress on gene expression patterns in different tissues of pearl millet. **Figure 5** illustrates the expression pattern of DEGs across all the pairwise combinations of HTG and HSG.

### Uniquely expressed genes and their regulation associated with photosynthesis and CO_2_ assimilation

Photosynthesis, photochemical reactions, chlorophyll biosynthesis, NADPH and ATP synthesis, and respiration are all vital physiological processes in plants that help them adapt to heat stress. However, photosynthesis becomes susceptible at high temperatures, with its components, particularly PS II, being extremely sensitive (Ashraf and Harris, 2013). This susceptibility reduces photosynthetic efficiency, limiting plant development. Our study discovered 347 genes associated with respiration and photosynthesis pathways, including 90 uncharacterized proteins. Heat stress drastically reduced the expression of genes involved in CO_2_ assimilation and photosynthesis in the sensitive than tolerant genotype (**Figure 5**).

Several vital genes involved in electron transport, chlorophyll production, and carbohydrate metabolism, including *chlorophyll-binding a/b proteins, PS I* and *PS II components, PsbP domain-containing protein, 2-hydroxy-acid oxidase, cytochrome P450, phosphoenolpyruvate carboxykinase (PEPC), phosphoglucomutase, stachyose synthase, phosphoribulokinase (PRK), malate dehydrogenase, phosphoglycerate kinase* and *ferredoxins*, were significantly more down-regulated under heat stress in the sensitive over the tolerant genotype (**Table 4**). Xu and Huang also reported that in response to drought, heat and combined stress, chlorophyll-binding proteins were down-regulated in both the tolerant and sensitive *Kentucky bluegrass* genotypes, but the level of expression in the tolerant was less suppressed than the sensitive genotype (Xu and Bingru, 2012).

**Table 4:** Expression pattern of selected differentially expressed genes operating in important functional pathways under heat stress.

Category	Gene	HTG_Control *vs* HTG_Treatment	HSG_Control *vs* HSG_Treatment	HTG_Treatment *vs* HSG_Treatment
Nutrient/water uptake	*Bidirectional sugar transporter SWEET*	4.28 ^R/^4.02^L^	-4.48 ^R^	4.28 ^R^
	*Potassium transporter*	-2.03 ^R^	-5.17 ^R^	3.30 ^R^
	*Phosphate transporter*	–	-4.58 ^R^	–
	*Glutamine synthetase*	–	-3.92 ^R^	–
	*ABC transporter*	4.50 ^L^	-6.97 ^L^	-5.12 ^R^
	*Copper transporter*	-2.13 ^L^	-5.48 ^R^	3.03 ^L^
	*Nitrate reductase*	–	-5.27 ^R^	-4.70 ^L^
Photosynthesis and CO2 assimilation	*Chlorophyll a-b binding protein*	-2.70 ^R^	-12.79 ^R^	8.23 ^L^
	*PS-I*	-3.56 ^L^	-12.57 ^R^	3.36 ^L^
	*PS-II*	-3.54 ^L^	-9.22 ^R^	2.95 ^L^
	*PsbP*	-2.22 ^L^	-7.82 ^R^	2.77 ^L^
	*PEPC*	-3.19 ^R^	-12.73 ^R^	3.01 ^R^
	*Glyceraldehyde-3-phosphate dehydrogenase*	3.60 ^L^	-9.64 ^R^	-4.38 ^R^
	*Rieske domain-containing protein*	-2.36 ^L^	-11.53 ^R^	5.16 ^L^
	*Phosphoribulokinase*	–	-5.65 ^R^	–
	*Phytocyanin*	3.39 ^L^	-7.07 ^L^	-6.51 ^R^
	*Carbonic anhydrase*	-2.41 ^L^	-8.22 ^R^	7.04 ^L^
	*RuBisCO*	-2.11 ^R^	-12.05 ^R^	2.19 ^L^
	*ATP synthase subunit alpha*	3.99 ^L^	-10.98 ^L^	-2.13 ^L^
	*Fructose-bisphosphate aldolase*	-2.61 ^R^	-12.55 ^R^	-3.27 ^R^
Secondary metabolites/ubiquitination genes	*Terpene cyclase*	-2.45 ^R^	-9.36 ^R^	2.99 ^L^
	*Clp*	2.73 ^L^	-3.13 ^R^	–
	*RING-type E3 ubiquitin transferase*	-2.81 ^R^	-5.03 ^R^	-2.22 ^L^
	*Lipoxygenase (LOX)*	-2.24 ^R^	-10.25 ^R^	-8.58 ^L^
	*Glyoxalase*	–	-4.05 ^R^	–
	*Lipase_3*	-3.02 ^L^	-10.32 ^L^	-2.28 ^L^
Signal transduction	*Protein kinase*	-2.43 ^L^	-6.02 ^L^	9.06 ^L^
	*MAPK*	-2.21 ^L^	-4.46 ^R^	–
	*AAI domain-containing protein*	3.90 ^R^	-11.95 ^R^	-4.34 ^L^
	*ARF*	3.19 ^L^	–	2.83 ^L^
	*MFS*	3.05 ^L^	-6.24 ^R^	7.95 ^L^
	*Cyclic nucleotide-gated ion channel*	–	-3.14 ^R^	–
	*Trehalose 6-phosphate phosphatase*	3.32 ^L^	–	-3.43 ^L^
ROS-Signaling	*POD*	3.62 ^L^	-6.75 ^L^	6.90 ^L^
	*CAT*	-2.04 ^L^	-4.69 ^R^	3.11 ^L^
	*Amine oxidase*	2.34 ^L^	–	-3.24 ^L^
	*Thioredoxin*	-2.59 ^L^	-9.03 ^R^	2.37 ^L^
	*Laccase*	-2.05 ^R^	-5.32 ^L^	-7.41 ^L^
Transcription Factors	*BHLH*	2.29 ^L^	-5.95 ^R^	7.95 ^L^
	*MYB*	–	-6.96 ^R^	3.67 ^L^
	*NAC*	5.79 ^L^	-5.59 ^R^	2.96 ^L^
	*WRKY*	3.02 ^L^	–	6.02 ^L^
Heat Shock proteins	*sHSPs*	8.73 ^L^	-5.02 ^R^	-3.65 ^L^
	*HSP70*	-2.07 ^R^	–	-4.57 ^R^
	*HATPase*	9.72 ^L^	2.24 ^L^	–

L- expression in leaf; R- expression in root

Genes encoding *phytocyanins* are pivotal in facilitating electron transfer exhibited induced expression in treated heat-tolerant genotypes. This upregulation suggests a potential role for *phytocyanins* in aiding stress adaptation mechanisms. The *Rieske* protein of cytochrome b6/f complex is a component of the photosynthetic electron transport chain in the chloroplast and was induced in the leaves of *Pinellia ternate* under heat stress (Zhu et al., 2013). The leaves of HTG recorded *Rieske* protein down-regulation to 2 folds. In contrast, the HSG was suppressed to -11 folds, indicating that the tolerant genotype under stress conditions adapts by conserving energy to cope with the adverse effects.

Under heat stress conditions, a significant decrease in gene expression related to photosynthesis and respiration pathways was evident. This decline was more pronounced in the sensitive genotype compared to the tolerant genotype (**Table 4**). This disparity suggests a higher susceptibility of sensitive genotypes to heat stress, resulting in more pronounced transcriptional suppression and a consequent reduction in respiratory processes. Heat affects the production of ATP and NADPH from the light reactions, and these, in turn, significantly affect the photosynthetic enzymes such as *RuBisCO*, *carbonic anhydrase* and *PRK*. *ATP synthase* α *subunit* expression was over-expressed in HTG and suppressed in HSG, which aligns with a study on *T. aestivum*, where *ATP synthase* α *subunit* activity was reduced in heat-sensitive and increased in the tolerant genotype (Wang et al., 2015). *Carbonic anhydrase* in *G. max* and *Agrostis species* was increased in the tolerant species than the sensitive ones under heat stress, suggesting it should play an important role in imparting tolerance (Xu and Huang, 2010; Das et al., 2016). Most of the genes involved in the photosynthesis process and CO_2_ assimilation under stress showed high connectivity with the significant hub genes (**Figure 3**).

**Figure 6:**
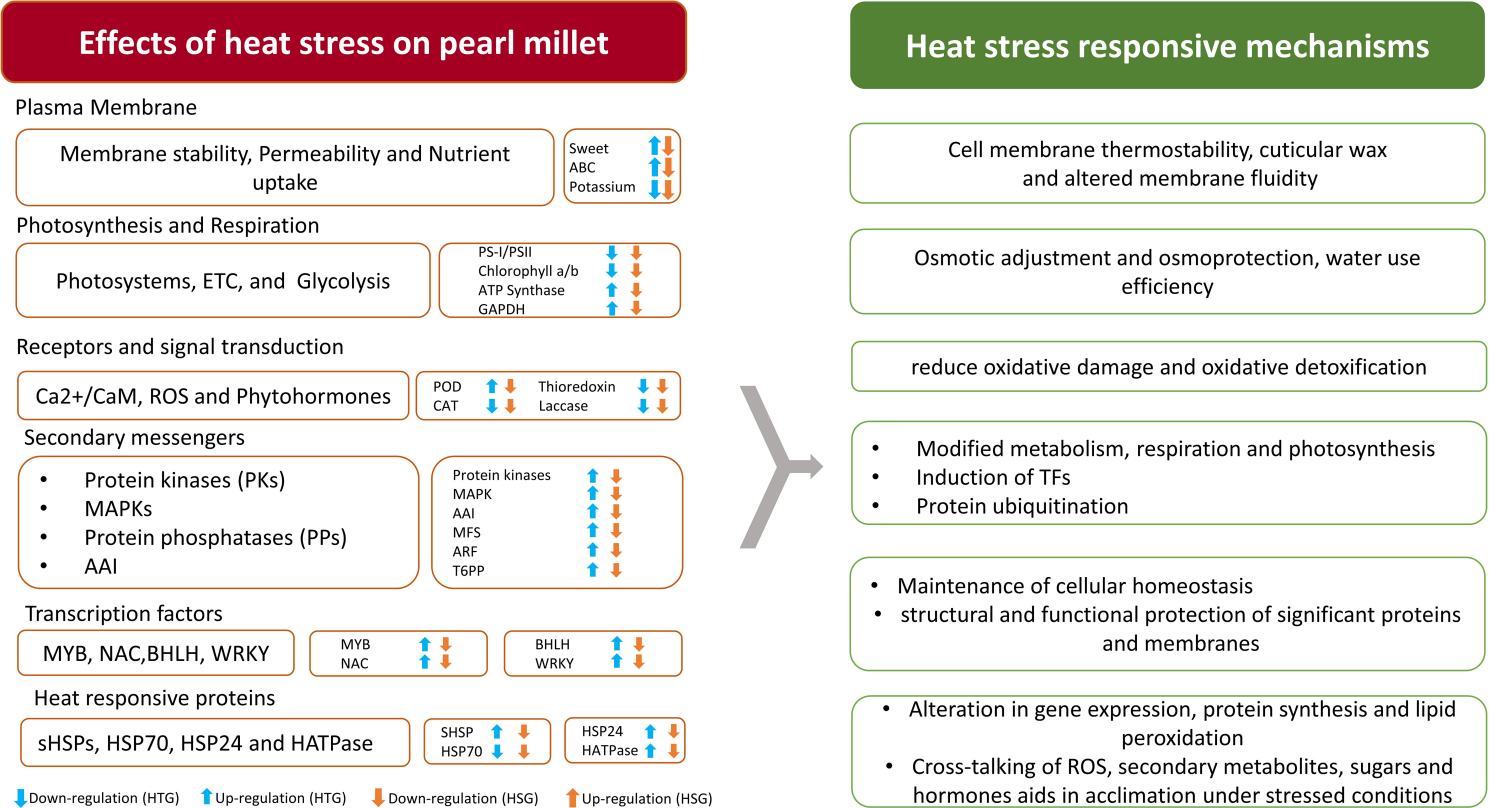
Elucidating the important genes operating in major pathways leading to heat stress tolerance in pearl millet.

The enzymes that play a key role in carbon flux in the Calvin cycle and in determining carbon assimilation, such as *PRK, glyceraldehyde 3-phosphate dehydrogenase (GAPDH) and fructose-bisphosphate aldolase (FBPA)*, were more suppressed in sensitive genotype than HTG (**Figure 6**). Previous studies have demonstrated the same results: *PRK* in rice*, A. stolonifera* and *A. scabra, GAPDH* in *A. stolonifera*, and *A. scabra* and *FBPA* in *G. max* and *M. sinensis* were suppressed under heat stress. Most of the genes involved in glycolysis and in the TCA cycle were down-regulated under heat stress in HSG, resulting in reduced respiratory electron transfer and oxidative phosphorylation in the sensitive than in the tolerant genotype. Similarly, genes involved in the Calvin cycle were also suppressed in the sensitive genotype. The tolerant genotype displayed less suppression of the above-mentioned genes in their leaves under heat stress conditions, indicating that respiratory carbon metabolism is significantly less inhibited under stress. This differential gene expression pattern favors the tolerant genotype, potentially aiding their adaptation to heat stress.

This observation aligns with previous findings (Kurimoto et al., 2004) and suggests that the genes responsible for glycolysis, the Calvin cycle, and the TCA cycle exhibit a more resilient expression profile in the leaves of the tolerant genotype, contributing to their better adaptation to heat stress by conserving carbon resources. Reduction in respiratory and photosynthetic activity is correlated to a decrease in grain yield. At the cellular level, heat stress generates ROS that disrupt chloroplast membranes and the plasma membrane, leading to photosystem deactivation, reduced photosynthesis, and *RuBisCO* inactivation (Takahashi et al., 2007). This hampers the production and allocation of photo-assimilates, affecting grain’s anthesis, filling, size, number, and maturity, ultimately reducing crop productivity. Genes associated with chloroplast, photosynthesis, photosystem II, plant-type cell wall, and chloroplast thylakoid membrane were considerably enriched based on GO enrichment.

### Investigating the impacts of heat stress on nutrient and water uptake genes

Plant growth and development depend on adequate nutrient and water availability, primarily controlled by roots via mineral cycling and phytohormone signaling (Freschet et al., 2018; Kong et al., 2012). Heat stress dramatically impairs the synthesis of proteins involved in the uptake and transportation of nutrients. Our research discovered critical gene expressions that regulate amino acids, carbohydrates, and vital micronutrients (e.g., zinc, potassium, magnesium, boron) and macronutrients (e.g., phosphate, copper) transport across different parts of a plant (Fahad et al., 2017; Zelazny and Vert, 2014).

The *bidirectional sugar transporter SWEET* promotes carbohydrate transport across membranes, influencing plant resistance to osmotic stress (Darko et al., 2019). Klemens (2013) demonstrated that over-expression of *AtSWEET16* in *Arabidopsis* improved osmotic and cold tolerance (Klemens et al., 2013). The *SWEET* gene showed up-regulation in the leaf and root tissues of HTG. It is involved in the regulation of abiotic stresses such as drought and heat in different crops (Yuan and Wang, 2013). Sensitive genotype roots displayed diminished nutrient absorption under heat stress, associated with reduced expression of *phosphate transporter*, *bidirectional sugar transporter SWEET*, and *copper transporters* in contrast to their up-regulation under control conditions (**Figure 6**). Similarly, in the roots of HSG, *potassium transporters* and enzymes involved in nutritional assimilation, such as *nitrate reductase* and *glutamine synthetase*, exhibited suppressed expression under stress conditions (**Table 4**). Potassium fluxes are essentially required to regulate the transpiration process, so the down-regulation of potassium transporter may affect the plant’s response to heat and oxidative stress (Mulet et al., 2023). Furthermore, *Rieske domain-containing proteins* (-11.5 FC) involved in metal ion binding and nitrate assimilation were significantly down-regulated, particularly in the heat-sensitive genotype (Molik et al., 2001).

The *ABC transporter domain-containing protein*, an integral membrane component involved in ATPase-coupled transmembrane transporter activity and ATP binding (Dahuja et al., 2021), was repressed in the roots of the heat-treated sensitive genotype and induced in the leaf of HTG. *ABC transporters* are involved in the regulation of various metabolisms, growth, development and environmental responses. ABC transporters in model plants such as *Arabidopsis* and rice have been identified to resist biotic and abiotic stress (Moon and Jung, 2014). The down-regulation of genes associated with nutrient uptake, primarily observed in the HSG combination, led to decreased nutrient uptake, resulting in reduced root and shoot biomass of sensitive than in tolerant genotype.

Aquaporin PIP2, a membrane channel protein in the roots of the tolerant genotype, contributed to ionic balance maintenance, which is essential for water and solute transport, and showed enhanced expression, assisting heat tolerance by regulating water balance (Maurel et al., 2008; Giri et al., 2017). Previous studies suggested that PIPs have a role in modulating plant root’s water intake and function in plant heat tolerance (Vandeleur et al., 2005). Obaid et al. (2016) discovered that over-expression of three AQP genes increased water utilization and induced heat tolerance in *Rhazya stricta* (Obaid et al., 2016). Efforts have been made over the last decade to understand the function of PIPs, and a few studies have demonstrated that PIP gene over-expression is favorable in imparting tolerance under heat-stress conditions. The GO study discovered novel genes involved in water channel function and cellular response to water scarcity, revealing the active engagement of diverse cellular components in nutrition and water intakes, such as chloroplast structures and sugar transporters. Kegg pathway analysis revealed that 39 genes participated significantly in carbon metabolism, aiding in combating heat stress by providing the energy required for the maintenance of metabolic and cellular responses.

### Genotype-specific responses of secondary metabolites biosynthesis and protein ubiquitination pathways

Our findings revealed unique gene expression patterns associated with 19 pathways corresponding to tolerant and sensitive genotypes, spanning critical biological activities. Pathways involved in amino acid, carbohydrate, photosynthesis, glycan, lipid, phenyl-propanoid metabolism, protein ubiquitination, and secondary metabolite production were significantly enriched. Notably, genes implicated in protein ubiquitination/modification pathways in the tolerant genotype, including *caseinolytic protease (Clp)* and *ring type E3 ubiquitin transferase*, displayed induced expression in the tolerant than sensitive genotype. *Clp* are chaperones involved in protein disaggregation; these proteases are required to protect against oxidative stress (Pulido et al., 2017). The PPI network revealed 39 ribosomal proteins that interacted with the key genes involved in the cellular response to heat stress (**Figure 3**). The intricate network of interactions between ribosomal proteins and stress-responsive genes explains the complex mechanisms that plants use to adapt and thrive under stress conditions (**Figure 6**). These findings are congruent with earlier research emphasizing varying crop responses to heat stress. High-temperature treatment affects the physiological functions of ER, hence affecting the protein’s synthesis, modification and proper folding.

Stress-modulated lipid metabolism, with *lipoxygenase* (LOX)*, lipase_3, lipase_GDSL domain-containing* and *patatin* enzymes were significantly more down-regulated in sensitive genotypes, indicating repressed lipid metabolic pathways (**Table 4**). *LOX* is documented in several crops to catalyze the synthesis of plant’s defense-related *9(S)-hydroperoxy-octa-deca-trienoic acid* (Göbel et al., 2001). In research on tomato seedlings, increased lipoxygenase activity was correlated with salt tolerance, and in pepper, the *CaLOX1* gene was reported to modulate the abiotic stress responses via activation of defense-related marker genes and scavenging of ROS (Lim et al., 2015). *Lipase_GDSL* plays an important role in plants’ defensive mechanism. For example, *AtLTL1* encoding *lipase_GDSL* in *Arabidopsis* was recorded to enhance salt tolerance (Naranjo et al., 2006).

Secondary metabolites have multifaceted roles in plant-environment interactions and provide pigmentation to various plant parts. However, *terpene cyclase, terpene synthase*, involved in the synthesis of metabolites that assist in the regulation of homeostasis and plant’s response to biotic and abiotic stress and *glyoxalase*, important in secondary metabolite biosynthesis, displayed less suppression in tolerant genotype while experiencing significant more down-regulation in sensitive genotype (Pérez-Llorca et al., 2023). Secondary metabolites in some plants function as osmolytes and growth precursors to help plants recover from heat stress.

Phenylpropanoids play a crucial role in lignin synthesis, particularly under high temperatures. This observation suggests a modulation in the production of compounds responsible for lignin synthesis, a key aspect of plant stress response, in the heat-tolerant genotype. The decrease in secondary metabolite biosynthesis in tolerant genotype at high temperatures might signify a strategic energy conservation response under high-temperature conditions. These changes in gene expression related to secondary metabolites and protein ubiquitination pathways point to a nuanced adaptive response to heat stress. These alterations emphasize the genotype-dependent regulation of significant stress adaptation pathways in pearl millet, reflecting the diverse strategies employed by different genotypes in coping with heat stress conditions. Kegg pathways represent the involvement of 180 genes in the biosynthesis of secondary metabolites which includes *3-ketoacyl-CoA synthase* displaying up-regulation and down-regulation in the control *vs* treatment comparison of tolerant and sensitive genotype, respectively.

### Increased temperature alters gene expression in signal transduction pathways

Elevated temperatures promote significant changes in gene expression within signal transduction pathways in plants, particularly when subjected to abiotic stress such as heat. These changes activate sophisticated regulatory networks, which trigger innate defense mechanisms. *Protein kinases*, particularly *MAPKs*, are important in orchestrating physiological adaptations by transducing environmental stimuli to the nucleus, thereby protecting plants from diverse biotic and abiotic stresses (Morrison, 2012). Furthermore, the *Cysteine-rich receptor-like protein*, a member of the RLK family, is involved in plant immunology, stress response, and growth and development (Tanaka et al., 2012). Importantly, elevated temperatures cause an increase in calcium influx, which is one of the first cellular alterations that occurs after a heat shock.

In this study, we found a significant down-regulation of genes encoding *MAPK*, *protein kinase* (-5.7 to -10.2 times), *ABC1 domain-containing protein* associated with protein kinase activity, *calmodulin-binding protein 60*, and *cysteine-rich receptor-like protein* in the sensitive genotype (**Table 4**). Heat stress triggers the *MAPK* signaling cascades in the plant system. *MAPK* is a master regulator that operates various physiological and cellular activities in response to heat stress. In *wheat*, *MAPK* triggers different genes that impart heat tolerance under terminal heat stress (Banerjee et al., 2020). This evidence indicates that heat stress has a more adverse impact on the expression of signaling genes in sensitive genotypes.

Integral membrane proteins, particularly *ABC transporter proteins* that use ATP as energy source, displayed reduced expression in most combinations except in tolerant genotype under stress conditions (**Figure 6**). However, membrane transport protein-encoding *MFS (Major facilitator superfamily)* genes, which facilitate compound transport across cell membranes using electrochemical gradients, were more down-regulated in various HSG combinations than in HTG, implying potential disruptions in membrane transport functions within leaves and subsequent effects on distinct cellular activities (Niño-González et al., 2019).

Further, *cyclic nucleotide-gated ion channels (CNGCs*), which are involved in calcium signal transduction, expression was repressed in the roots of the heat-sensitive plants under stress, similar to the findings in *Arabidopsis* (Alqurashi et al., 2016). This down-regulation is consistent with previous findings in *Arabidopsis* seedlings, implying a negative impact on thermo-tolerance by increasing ROS enzyme activity (Gao et al., 2012). Additionally, the observed suppression of gene expression for *trehalose 6 phosphate phosphatase (T6PP)* in sensitive genotype under stress conditions suggests a plausible reduction in trehalose levels induced by heat stress, potentially disrupting carbohydrate transport mechanisms critical for stress tolerance (Lunn et al., 2006; Ruan, 2014). The tolerant genotype displayed induced expression of *T6PP* in leaf compared to HSG. *T6PP* acts as a sugar signal and induces the expression of genes associated with stress injury (Lyu et al., 2018).

Plant growth regulators, also known as phytohormones, are essential in reacting to abiotic stress, particularly heat stress. Recent studies reveal that hormones such as auxin, cytokinin, ethylene, and abscisic acid (ABA) are actively implicated in heat response. *ABA*, a crucial stress-related hormone, and its association with the up-regulation of *AAI (an abscisic acid-inducible protein)* across the combinations of the tolerant genotype imply its potential involvement in conferring heat stress tolerance (**Table 4**). ABA boosts tolerance by regulating the transcript level of HSPs and engaging in spatial and temporal interactions with ROS (Suzuki et al., 2016). *Auxin*, responsible for cell wall synthesis and nucleic acid metabolism, activates multiple genes involved in auxin-mediated signaling pathways, including *ARF, auxin efflux carriers*, and *short auxin-up RNA (SAUR*) (Fenqi et al., 2023). The research discovered a two-fold increase in *ARF* gene expression in the stressed leaves of the tolerant genotype. In the heat-sensitive genotype, the *SAUR 36* gene, which is related to leaf senescence and cell elongation suppression, was down-regulated (Jia et al., 2020).

Earlier findings revealed complex interactions between ethylene, ABA, and brassinosteroids in regulating heat stress responses. We identified the activation of *CASP* proteins and 19 undiscovered proteins associated with the brassinosteroids signaling pathway. Heat stress decreases cytokinin synthesis, influencing cell division and elongation, with down-regulation recorded in genes involved in cytokinin biosynthesis and *zeaxanthin epoxidase*, a key player in hormone synthesis (Hoang et al., 2020). The suppression was more pronounced in sensitive genotype under heat stress. These findings suggest that the tolerant genotype employs distinct adaptive mechanisms in response to heat stress compared to the sensitive genotype.

### ROS scavenging: a key pathway for modulating heat stress response mechanisms

When exposed to high temperatures, plants overproduce ROS, a pivotal signaling component that causes oxidative stress by destroying the cell structure, particularly the membrane structure (Slimen et al., 2014). Plants have ROS scavenging strategies to mitigate the damage, essential for cellular recovery and redox equilibrium (Kotak et al., 2007). In this study, we noticed differences in the expression of ROS-scavenging enzymes in response to heat stress in both genotypes.

Several ROS scavenging genes namely, *superoxide dismutase (SOD), ascorbate peroxidase (APX), catalase (CAT), peroxidase (PRX), glutathione peroxidase (GPX), amine oxidase, amino oxidase, respiratory burst oxidase, thioredoxin, and glutaredoxin* activated under the stress condition (**Figure 6** **).** We discovered 52 *peroxidase-related* genes involved in suberin and lignin synthesis, stomatal closure control, and stress-induced heat shock protein (HSP) expression (Havaux, 1993). This enzyme is involved in scavenging ROS, which are produced in response to heat stress. *Peroxidase* gene expression was significantly suppressed in the HSG treated conditions, and in the leaf of tolerant genotype under stress, it displayed induced expression (**Table 4**). In the roots, *SOD*, which is important for dismutating superoxide radicals (O_2-_) into oxygen (O_2_) and hydrogen peroxide (H_2_O_2_) and thereby preserving photosynthetic organelles, was down-regulated in HSG under stress (Zang et al., 2020). Over-expression of *SOD* in *Avicennia marina* and *Alfalfa* confers tolerance to abiotic stress (Rubio et al., 2002; Prashanth et al., 2008).

*Amine oxidase*, involved in quinone binding and amine metabolism, influences plant responses to environmental stress and demonstrated induced expression patterns across tolerant genotype combinations (Gholizadeh and Mirzaghaderi, 2020). *APX*, which regulates hydrogen peroxide levels under heat and oxidative stress, displayed varying expression patterns across the tissues in both genotypes. The expression of *APX* in *Arabidopsis* resulted in chloroplast protection during heat stress (Panchuk et al., 2002). DEGs encoding *APX* were primarily more down-regulated in the sensitive than the tolerant genotype, indicating that in response to heat stress, HTG over-produced ROS, resulting in increased APX activity. The down-regulation of these genes suggests a possible disruption of metabolic adjustments in the leaf and roots of *pearl millet* under heat stress, primarily in the sensitive genotype.

Genes related to ROS detoxification, including *thioredoxin* involved in ROS signaling, *respiratory burst oxidase*, *CAT, GPX*, and *laccase*, which contributes to oxidoreductase activity in the apoplast, displayed down-regulation predominantly in the sensitive genotype under stress conditions (Mittler, 2017). This down-regulation indicates heightened oxidative stress in sensitive genotypes. In contrast, the tolerant genotype exhibited reduced oxidative stress despite the down-regulation of these genes. This observation aligns with recent studies that reported decreased expression of ROS-scavenging genes under heat stress. GO demonstrated a significant representation of 60 oxidative stress-related genes in the category of oxidoreductase activity, highlighting the importance of ROS buildup on plant responses.

### Role of transcription factors in heat stress resilience

**Figure 7:**
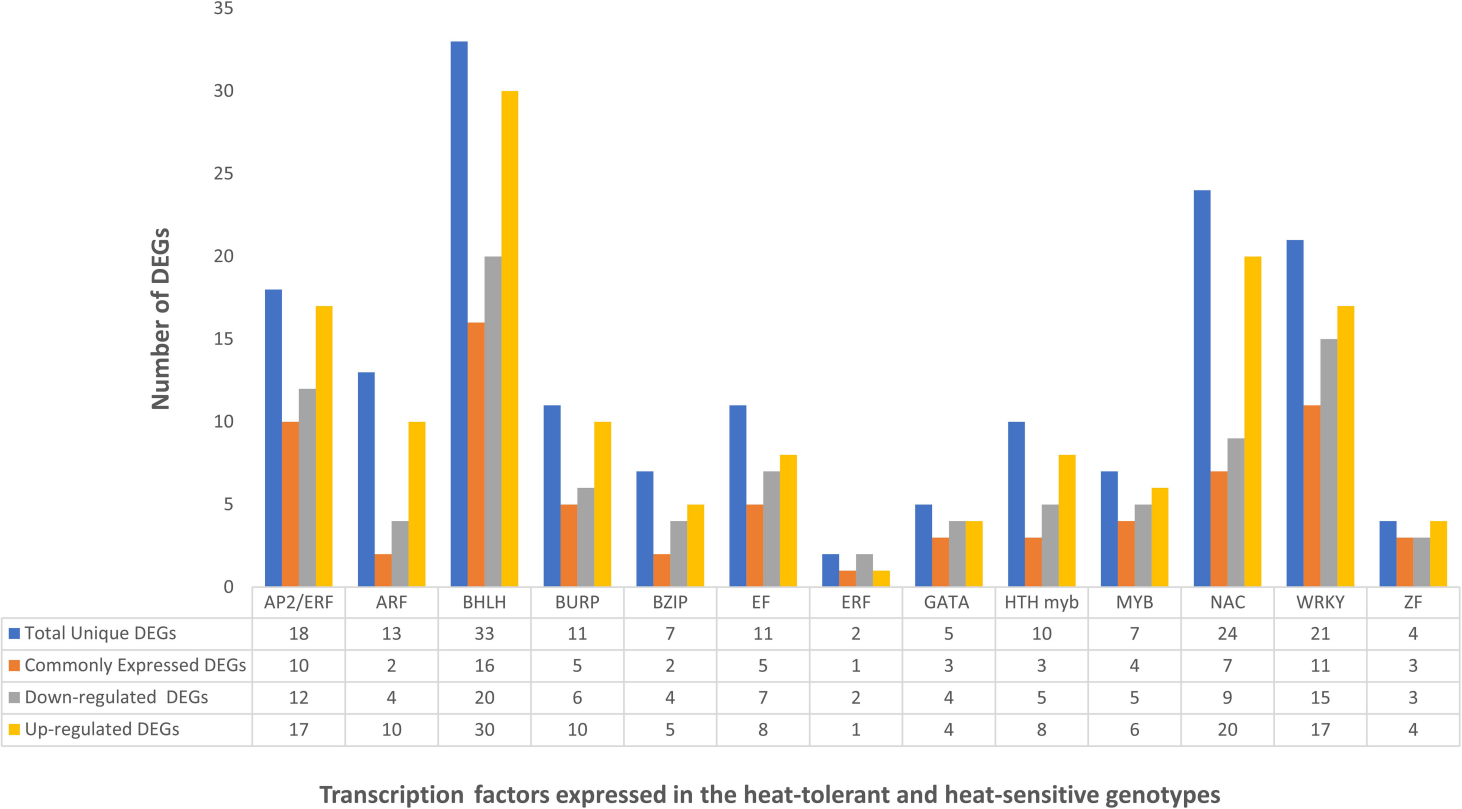
Expression of important transcription factors in the leaf and root tissues of heat-tolerant and heat-sensitive genotypes.

TFs are essential in regulating transcriptional responses to heat stress, and various TFs implicated in heat stress acclimation were found in multiple crops. Previous studies have reported the differences in the expression of different TF families, including *WRKY, NAC, AP2/ERF, MYB, EF, GATA, bZIP, MADS-box, DEAD-box ATP-dependent RNA helicase, zinc finger protein, C2H2*, and *C3H domain-containing protein*, highlighting their role in the heat stress response (Ward and Schroeder, 1994; Seki et al., 2003). **Figure 7** represents the expression of different TFs across the combinations of HTG and HSG. Among the identified TFs, *MYB*, a significant player in chromosomal structure and stress interactions and is involved in the biosynthesis of secondary metabolites, exhibited distinct expression patterns (Samad et al., 2017). El-Kereamy et al., 2012 found that heat stress triggered the activation of *OsMYB55* (El-Kereamy et al., 2012). Overexpressing *OsMYB55* alleviated the adverse effects of high temperatures on grain yield by enhancing amino acid metabolism and improving rice’s heat stress tolerance. Seventeen differentially expressed *MYB* genes displayed reduced activity (**Table 4**) primarily in leaf and root tissues of the sensitive genotype, but upregulation in the tolerant genotype, indicating a vital function in regulating transcription under heat stress conditions (Chakraborty et al., 2022).

The *C2H2* TF family and a single *C3H* gene, implicated in growth, development, and abiotic stress responses, showed different expression profiles (Thirunavukkarasu et al., 2017; Hu et al., 2019). *WRKY, NAC, AP2/ERF*, *BHLH* and *BURP* genes were significantly up-regulated in the tissues of the tolerant genotype (**Table 3**). In contrast, the expression of *bZIP, MADS-box, BHLH, DEAD-box ATP-dependent RNA helicase, EF*, and *zinc finger* proteins were drastically reduced in sensitive genotype leaf and root tissues. The role of *WRKY* in regulating gene expression during heat and drought stress is consistent with our findings, indicating their potential for improving heat stress tolerance in *Arabidopsis* and *rice* (Wu et al., 2009; He et al., 2016). Similarly, enhanced expression of *NAC* and *AP2/ERF* in the tolerant genotype supports the finding of the earlier research demonstrating their significance in imparting tolerance to multiple abiotic stresses across many crops (Sakuma et al., 2002; Nakano et al., 2006; Shiriga et al., 2014). The *AP2/ERF* family, comprising plant-specific TFs, possesses a conserved DNA-binding domain. This family contains DRE-binding proteins that activate stress-responsive genes by specifically binding to the dehydration-responsive element/C-repeat (DRE/CRT) in gene promoters (Sakuma et al., 2002; Nakano et al., 2006). The *AP2-ERF* super-family significantly influences plant growth, development, hormonal regulation, and responses to diverse environmental stresses, notably heat stress (Mizoi et al., 2012). *SNAC3* in rice enhances the tolerance to heat and drought stress by modulating the ROS balance (Xi et al., 2022). A study on *switchgrass* showed that *DEAD-box ATP-dependent RNA helicases* may function as RNA chaperons (Li et al., 2013).

Moreover, the over-expression of *BURP* in the leaf of tolerant genotypes implies their participation in plant hormone signaling and adaptation to environmental stressors (Shu et al., 2018). In alignment with previous research, the down-regulation of *bZIP* and *zinc finger* protein expression in sensitive genotypes indicates that heat stress has a negative impact on growth and many signaling pathways. In the reproductive stage of *Arabidopsis*, *bZIP* regulates heat tolerance (Gao et al., 2022). *BHLH* play diverse roles in plant development and stress responses. The significant inhibition of *BHLH* gene expression in sensitive genotype’s leaf and root tissues suggested that it plays an essential role in regulatory networks responding to heat stress. Studies revealing the drought-responsive behavior of *bHLH* genes, such as *MdbHLH130* in apples, and their participation in strengthening plant resistance highlight their potential significance in heat stress response pathways (Guo et al., 2021).

A study conducted on foxtail millet reveals the involvement of numerous *bHLH* genes in promoting drought tolerance, and *Fe2OG dioxygenases* are involved in various metabolic processes (Wang et al., 2018). Enrichment analysis of the TFs revealed that the TF-associated GO terms provide valuable insights into the underlying regulatory mechanisms and offer targets for further functional studies to elucidate their specific roles in plant stress response and development. Our research highlighted the conservation and diversity of TFs involved in heat stress response across various crops, and found that the foxtail millet is closely related to pearl millet TFs (**Figure 4**). The differential expression of TFs in both genotypes’ leaf and root tissues suggests that they play an important role in regulatory pathways and transcriptome reconfiguration during heat stress in pearl millet. It implies that these TFs have a complicated interplay in the plant’s response to heat stress, necessitating additional research into their precise regulatory roles.

### The elevated response of heat shock proteins and heat shock factors in pearl millet under stressed conditions

**Figure 8:**
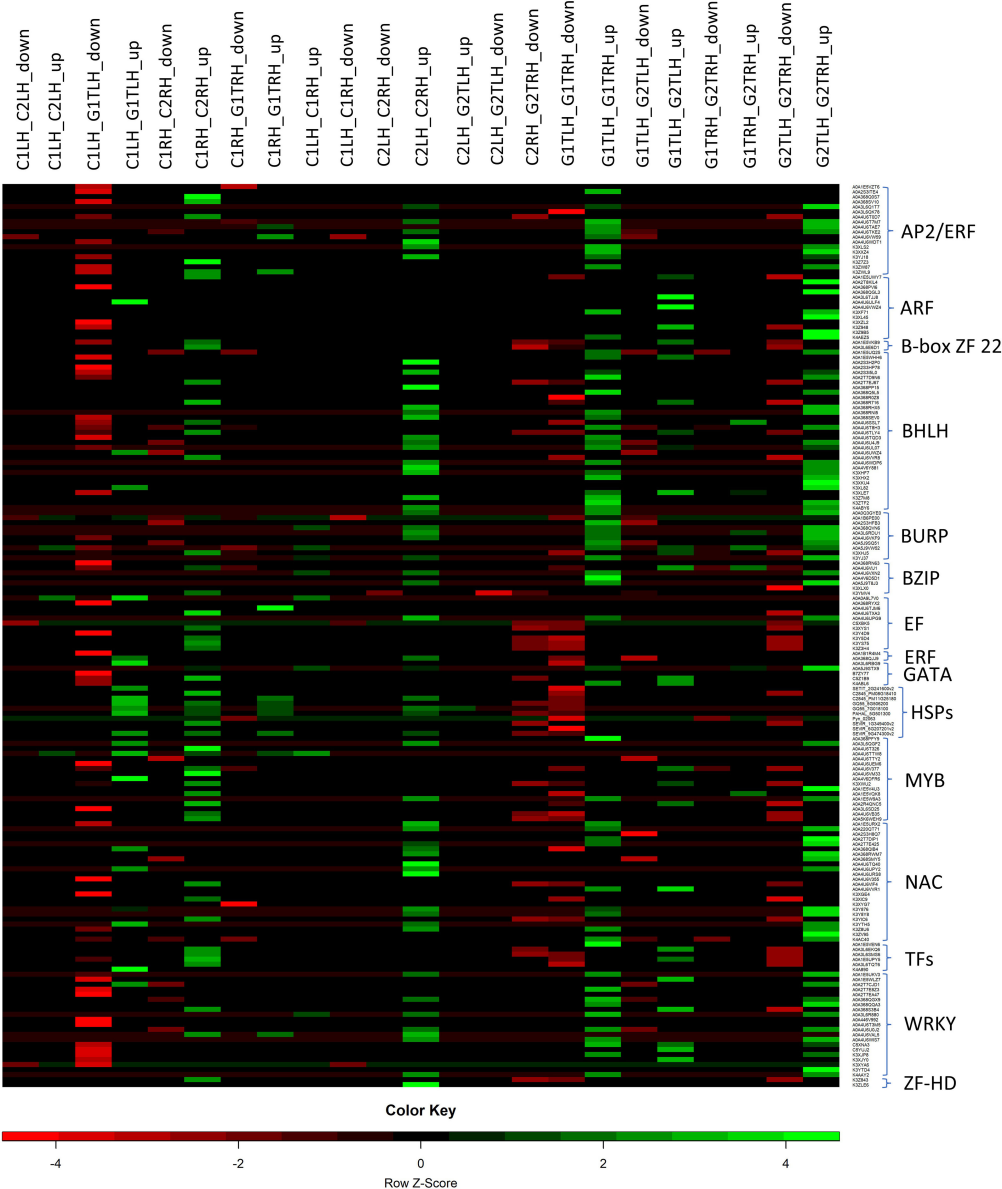
Heatmap of the selected TFs and HSPs operating under control and heat stress conditions in leaf, root and between leaf and root combinations of HTG and HSG. C1 and G1: HTG control and treatment conditions, C2 and G2: HSG control and treatment conditions, LH: leaf and RH: root, up: up-regulation and down: down-regulation.

Heat stress affects plant cell membrane integrity, alters protein structure, causes misfolding of native proteins, and promotes the accumulation of aberrant proteins. Plants have evolved different defense mechanisms in response to heat stress, including generating heat stress factors critical in regulating HSPs (Mittler et al., 2012). These HSPs function as molecular chaperones essential for maintaining cellular homeostasis. Several heat-related genes (**Figure 6**), including *HSP70, HSP24, HSP10kDa, sHsp17.6, sHSP domain-containing protein, sHsp17.0, HSF domain-containing protein*, and *HATPase_c domain-containing protein* were over-expressed. **Figure 8** represents the expression pattern of HSPs and TFs across the pairwise comparisons of HTG and HSG. When exposed to heat, pearl millet synthesize stress proteins, specifically HSPs, which act as molecular chaperones, promoting protein folding and structural stability (Mukesh Sankar et al., 2021).

Our findings revealed significant up-regulation of *sHsp17.0, sHsp17.6, and sHSP* domain-containing proteins in the leaf and roots of the tolerant genotype (**Table 4**). The expression of *sHsp17.0* was prominently observed in root tissues of HTG under stressed conditions, and sHsp17.0 exhibited significant up-regulation in roots of treated conditions in the tolerant genotype (**Figure 8**). Several studies in *Arabidopsis, P. pastoris*, and woody plants have demonstrated that sHSPs confer heat and drought tolerance (Yang et al., 2017; Wang et al., 2017). Additionally, *sHSP* domain-containing proteins displayed up-regulation in the leaf and root tissues of HTG. The enhanced activity of HSP70, which is prevalent under stress, was significant, with 7*0kDa stromal HSPs* expressing in both HSG and HTG roots under stress, indicating a more significant transcriptional response in roots compared to leaves.

*Stromal 70kDa HSP* participates in transport pathways between the endoplasmic reticulum, mitochondria, and chloroplastic pre-protein intake. According to previous research on various plants, including *Hevea brasiliensis*, rice, tobacco, and wheat, *HSP70* plays an important role for cellular response to heat stress (Zhang et al., 2009; Wang et al., 2016; Wahab et al., 2020). HSPs in pearl millet, particularly *HSP70* and *HSP90*, have been studied for their role in heat and drought adaptation processes (Donald et al., 2015). It has been found that *HSP70* is involved in molecular chaperone activity under stress and plays a function in photo-protection and photosystem II repair (Schroda et al., 1999; Sung et al., 2001). *HATPase*, a member of the *HSP90* family, showed over-expression in tolerant genotype combinations, signifying its function in ATP binding and *ATPase* activity (**Figure 8**). Five genes related to *HATPase* were identified in soybean and rice, with the majority exhibiting up-regulation in the tolerant genotype combination (Schroda et al., 1999; Sung et al., 2001; Li et al., 2020). A comparative analysis of genes related to HSPs in pearl millet revealed orthologue sequences in rice, foxtail millet, sorghum, proso millet, and maize (**Figure 4**). This suggests that these genes are expressed in response to stress in various crops. The prevalence of HSPs, especially across HTG genotype combinations, highlights their crucial role in maintaining protein structure, ATP binding, and hydrolysis in response to heat stress and raising heat stress tolerance.

HSPs and heat-related genes are also regulated by heat shock transcription factors (HSFs), which orchestrate the plant’s response to heat stress (Guo et al., 2016). Our findings revealed that HSF-related genes were up-regulated in tolerant genotypes and down-regulated in sensitive cultivars. Zhu et al., 2006 showed that over-expression of *GmHSFA1* in soybeans confers thermo-tolerance, possibly due to the activation of *sHSP and HSP70* (Zhu et al., 2006). This implies that they play an important role in tolerant genotype survival under heat-stress conditions. The PPI network reveals that the expression of HSP is related to different important TFs and HSF in response to heat stress (**Figure 3**). The prominent expression of HSPs and HSF-related genes in the tolerant genotype emphasizes their critical engagement in cellular and molecular processes during heat stress, which aligns with recent research across many crops. The pattern of HSP expression in pearl millet under stress conditions supports previous research on heat-induced HSPs, emphasizing their critical role in plants’ response to heat stress.

Our research revealed substantial differences in the expression of genes involved in various physiological and biochemical processes, specifically in the tolerant genotype. We found significant differences in the expression of genes crucial in water, nutrient, and ion transport (*bidirectional sugar transporter SWEET*, *ABC transporter domain-containing protein, PIP, potassium transporters*, *nitrate reductase* and *glutamine synthetase)*, cell wall maintenance (*expansin)*, photosynthesis (*RuBisCO*, *carbonic anhydrase* and *ATP synthase* α *subunit)*, transcription factors (*BZIP, BHLH, MYB, AP2/ERF, and BURP)*, signal transduction (*protein kinases, MAPK, ABA*, and *AAI)*, ROS scavenging (*SOD, peroxidases, GPX, and amine oxidase)*, HSPs (*SHSPs, HATPase, and HSP 10kDa)*, secondary metabolite biosynthesis (*LOX, Lipase_GDSL*, *terpene cyclase, terpene synthase*, and *3-ketoacyl-CoA synthase)* and protein modification pathways (*Clp* and *ring type E3 ubiquitin transferase)*, implying that pearl millet regulates the synthesis and expression of these genes to survive under heat stress.

## Conclusions

A genome-wide transcriptome study was performed in two pearl millet genotypes (HTG and HSG) to examine the molecular mechanisms in response to heat stress. We comprehensively analyzed the leaf and root samples in three categories: between genotypes, within genotypes, and between tissues. These DEGs from pairwise comparisons of the treated and control samples provide substantial insight into the effects of heat stress on pearl millet.

The analysis of heat-responsive genes and hub genes revealed that protein processing in the endoplasmic reticulum is one of the critical pathways involved in heat stress. The protective impact of HSPs could be related to the chaperone mechanism network, in which multiple chaperones work together. Under stress, many structural proteins undergo adverse structural and functional modifications. As a result, refolding of denatured proteins and maintaining their function is crucial for cell survival in stress conditions. These findings could help in our understanding of the role of the genes implicated in heat tolerance.

We discovered a significant number of DEGs belonging to uncharacterized proteins, revealing possible new genes involved in heat stress response in pearl millet. Our study provides fresh insights into the transcriptional modifications in different tissues of tolerant and sensitive genotypes, which aids in decoding the underlying mechanism to enhance crop resilience. This study will set the groundwork for identifying and utilizing key genes that are explicitly expressed in tissues to investigate the mechanisms of heat stress tolerance in pearl millet. These genes will also serve as appropriate molecular indicators for screening accessions for heat tolerance and speed up the variety of development programs in pearl millet and similar millet crops.

## Data Availability

The data presented in the study are deposited in the NCBI Sequence Read Archive database with accession number PRJNA1062049.
