# Supplementary material for: Identification of key genes and molecular pathways regulating heat stress tolerance in pearl millet to sustain productivity in challenging ecologies
Source: Front Plant Sci. 2024 Aug 22;15:1443681. doi: 10.3389/fpls.2024.1443681 (PMC11374647; doi:10.3389/fpls.2024.1443681)
Supplement: Supplementary file 2 [file Presentation1.pptx]

## Slide 1
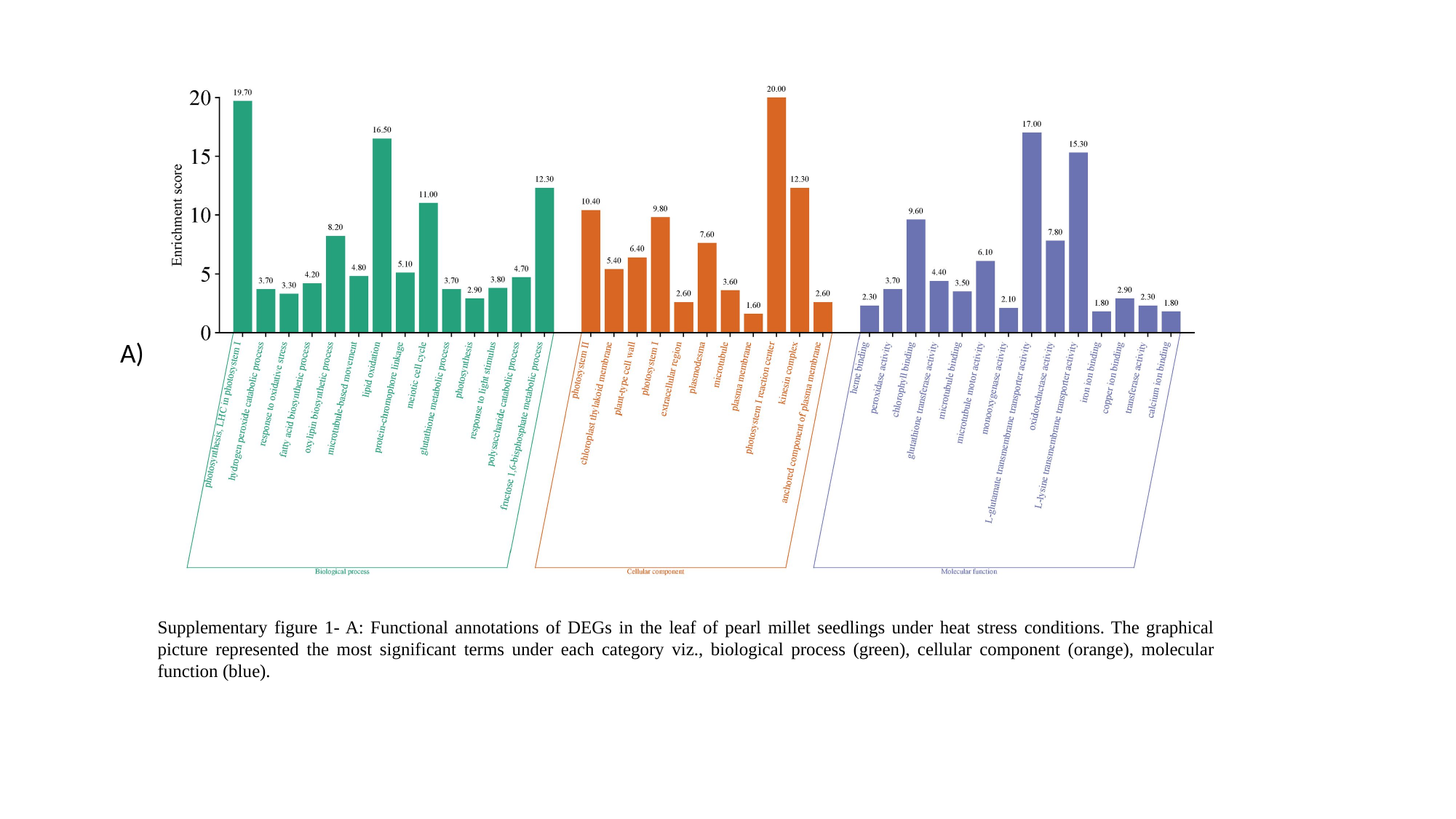

A)
Supplementary figure 1- A: Functional annotations of DEGs in the leaf of pearl millet seedlings under heat stress conditions. The graphical picture represented the most significant terms under each category viz., biological process (green), cellular component (orange), molecular function (blue).

## Slide 2
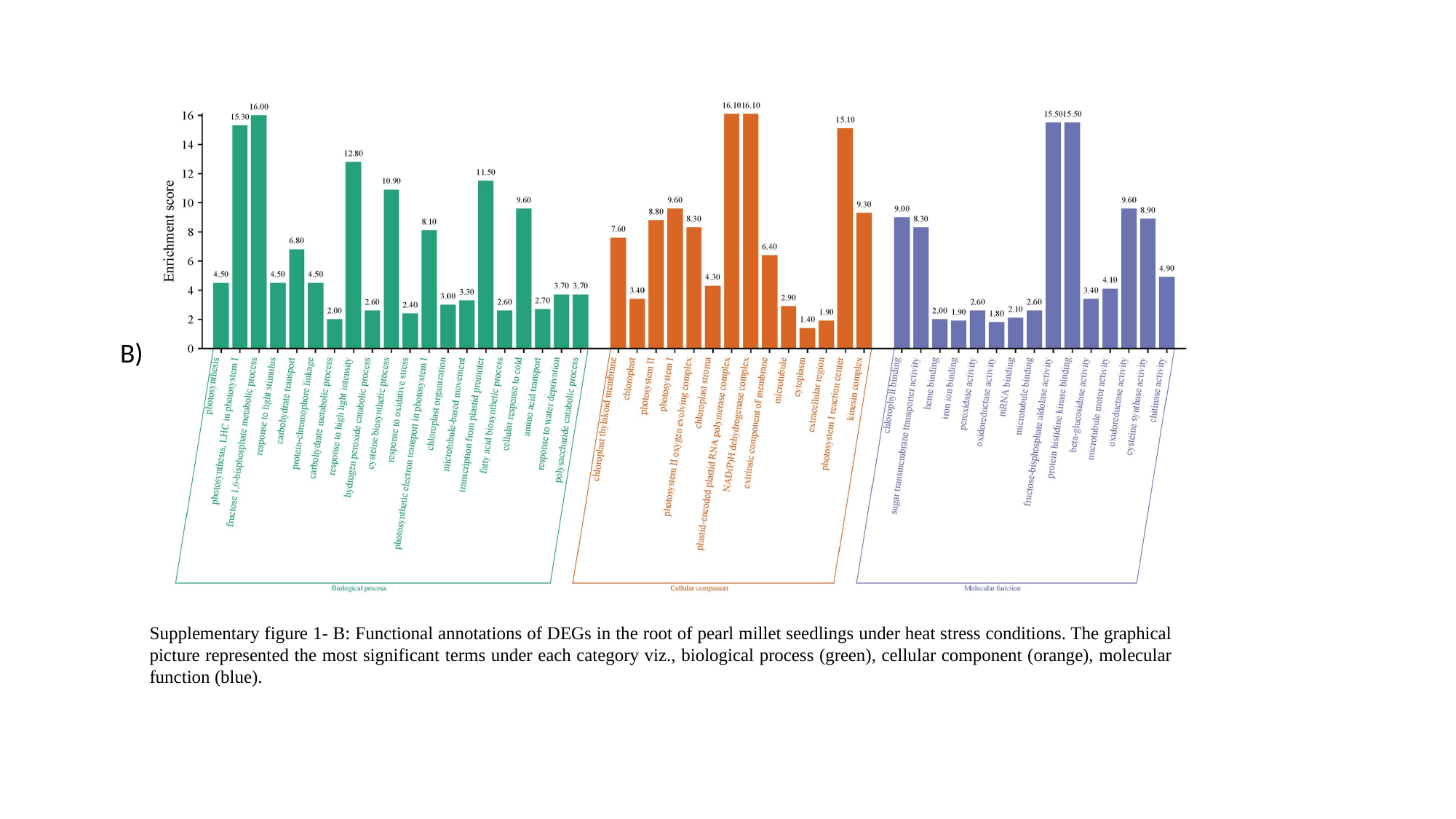

B)
Supplementary figure 1- B: Functional annotations of DEGs in the root of pearl millet seedlings under heat stress conditions. The graphical picture represented the most significant terms under each category viz., biological process (green), cellular component (orange), molecular function (blue).

## Slide 3
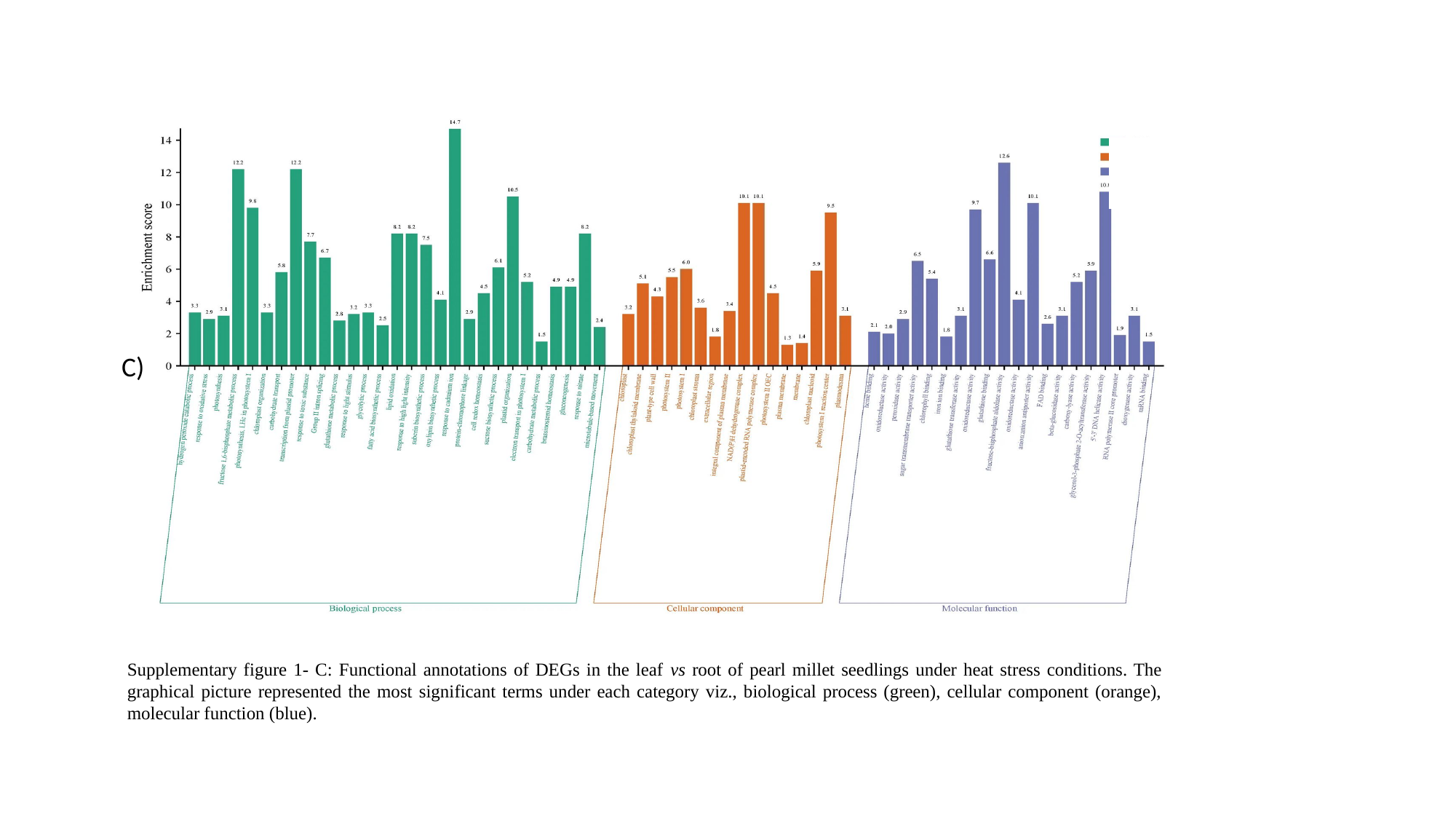

C)
Supplementary figure 1- C: Functional annotations of DEGs in the leaf vs root of pearl millet seedlings under heat stress conditions. The graphical picture represented the most significant terms under each category viz., biological process (green), cellular component (orange), molecular function (blue).
